# Efficacy of Internet-Based Self-Help Interventions for Irritable Bowel Syndrome: Systematic Review and Meta-Analysis of Randomized Controlled Trials

**DOI:** 10.2196/87216

**Published:** 2026-05-21

**Authors:** Xiaohong Xu, Ting Liu, Fang Wang, Hongyue He, Jiao Chen, Jingxian Liu

**Affiliations:** 1 College of Nursing, Chengdu University of Traditional Chinese Medicine, Chengdu, Sichuan, China; 2 Dean’s Office, Guang'an Hospital of Traditional Chinese Medicine, No. 1 Cuiping Road, Guang'an District, Guang'an City, Guang'an, Sichuan, China, 86 1-898-088-0156

**Keywords:** digital health, irritable bowel syndrome, self-help behavior, cognitive behavioral therapy, meta-analysis

## Abstract

**Background:**

Irritable bowel syndrome (IBS) is a common functional gastrointestinal disorder that reduces quality of life and causes a heavy medical burden. Internet-based self-help interventions are flexible and scalable, showing potential for IBS symptom improvement, but relevant evidence is fragmented and lacks systematic review.

**Objective:**

This systematic review aimed to comprehensively evaluate the effects of internet-based self-help interventions on symptom severity, quality of life, and visceral sensitivity, as well as comorbid depressive and anxiety symptoms, in individuals with IBS.

**Methods:**

Only randomized controlled trials evaluating internet-based self-help interventions for individuals with IBS were included. A literature search was conducted across PubMed, Embase, Web of Science, CINAHL Complete, PsycINFO, the Cochrane Library, and 4 Chinese databases on June 25, 2025, with an updated search on March 9, 2026. Risk of bias was assessed using the Cochrane Risk of Bias 2.0 tool. Meta-analyses were performed with the Hartung-Knapp-Sidik-Jonkman-adjusted random-effects model. Effect sizes were reported as standardized mean differences (SMDs) with 95% CIs, and evidence certainty was evaluated using GRADE (Grading of Recommendations Assessment, Development, and Evaluation) criteria.

**Results:**

A total of 17 randomized controlled trials from 7 countries were included, involving 2289 participants (predominantly female). Compared with control groups, internet-based self-help interventions were associated with a statistically significant improvement in IBS symptom severity (SMD −0.52, 95% CI −0.78 to −0.26, 95% prediction interval [PI] −1.46 to 0.42), quality of life (SMD 0.57, 95% CI 0.23 to 0.90, 95% PI −0.41 to 1.54), visceral sensitivity (SMD −0.55, 95% CI −0.89 to −0.21, 95% PI −1.43 to 0.33), and depressive symptoms (SMD −0.14, 95% CI −0.27 to −0.01, 95% PI −0.27 to −0.01). However, no statistically significant improvement was observed in anxiety symptoms (SMD −0.03, 95% CI −0.34 to 0.27, 95% PI –0.78 to 0.71). The certainty of evidence was rated as moderate to very low for all outcomes.

**Conclusions:**

This review synthesizes the latest evidence on internet-based self-help interventions for individuals with IBS disease-specific and comorbid psychological symptoms. It stands out by encompassing a diverse range of such interventions and incorporating visceral sensitivity as a key outcome. In doing so, it establishes a more comprehensive multi-outcome evidence base for IBS digital interventions, advancing the field by clarifying the potential of these interventions as viable alternatives to conventional treatments. For real-world practice, these findings can inform targeted strategies for primary care and telemedicine platforms, especially in resource-limited regions. However, this review is limited by moderate bias risk, high heterogeneity, and moderate to very low GRADE evidence certainty. A wide 95% PI suggests that effect variability is linked to contextual and population factors, so findings should be interpreted cautiously. Future research should prioritize technical support, patients’ digital health literacy, and standardized intervention protocols to further validate clinical utility.

## Introduction

Irritable bowel syndrome (IBS) is a common chronic gastrointestinal disorder affecting 5%‐11% of adults globally [1]. Its hallmark features include recurrent abdominal pain, bloating, altered bowel habits, and stool trait changes, often with urgency or incomplete evacuation [2]. Beyond physical discomfort, IBS is strongly linked to psychological conditions like anxiety, depression, hypochondriasis, and somatization [3,4]. A meta-analysis shows individuals with IBS have nearly 9 times the risk of depression compared with healthy individuals [5], with 28.8% experiencing depression and 39.1% anxiety [6]. The severity of these psychological effects is underscored by approximately 38% of individuals contemplating suicide [7].

These mental health challenges are linked to increased mortality rates and heightened susceptibility to disease [8]. Moreover, IBS significantly impairs patients’ quality of life and social functioning, imposing heavy societal burdens [1]. A 2015 survey by the American College of Gastroenterology found that IBS symptoms cause an average of 9 days of reduced productivity and 2 days of work absence monthly due to illness [9]. In the United States alone, annual indirect economic losses from IBS, due to work absenteeism and reduced productivity, are estimated at up to US $20 billion [10]. Thus, effective management to alleviate IBS symptoms, especially by addressing psychological issues, is critical.

A large proportion of individuals with IBS experience significant psychological distress or comorbid mental health disorders [11]. Mounting evidence points to a bidirectional relationship between psychological comorbidities and gastrointestinal function [12-14]. Though the exact pathogenesis of IBS is not fully understood, it can be framed via a bio psycho social model: psychosocial factors regulate physiological functions through the brain-gut axis, inducing somatization symptoms [15]; conversely, gastrointestinal pathological changes affect the central nervous system, worsening psychological symptoms [16].

Presently, psychological interventions like cognitive behavioral therapy (CBT), gut-directed hypnotherapy, stress management, and mental health education are proven to positively influence intestinal symptoms and the psychological state of individuals with IBS [17]. UK National Institute for Health and Care Excellence guidelines recommend considering referral to psychological interventions for those with persistent symptoms unresponsive to 12-month pharmacological treatment [18]. However, such therapies face challenges in clinical application. For example, psychological interventions involve high costs and poor patient compliance [19]. Moreover, reliance on professionals leads to limited accessibility [20], which is particularly notable in remote areas with scarce resources. Additionally, individuals with IBS with recurrent symptoms and psychological comorbidities need ongoing nursing support, but clinical care is constrained by human resources and follow-up cycles, making it hard to provide standardized, high-frequency symptom management guidance, resulting in insufficient patient self-management capacity.

Given the limitations of traditional intervention models and individuals with IBS’s need for self-management, symptom self-help strategies have become a key focus in clinical treatment. Self-help intervention, a self-driven approach targeting psychological issues, is part of standardized psychological therapy [21]. It uses diverse therapeutic resources with or without therapist guidance and enables individuals to learn independently for in-depth understanding and effective management of psychological issues [22]. Research by Dorn et al [23] shows that various self-help approaches benefit individuals with IBS symptoms and psychosocial status. As a nonpharmacological therapy, it helps individuals manage their condition and reduce health care costs through existing resources [23].

The delivery formats for self-help interventions have diversified, including internet-based platforms, mobile messages, apps, and printed materials [24]. Among these, internet-based formats have gained significant attention for their wide reach and accessibility. The global mHealth market hit a valuation of US $85.2 billion worldwide in 2024, and is expected to grow to US $284.83 billion by 2033 [25], driving the advancement of internet-delivered self-help interventions. These models fall into 2 categories: fully self-directed (providing automated standardized feedback and monitoring) [26,27] and low-intensity therapist contact (involving web-based encouragement and guidance) [28,29]. They boast advantages like low cost, privacy protection, easy accessibility, and convenience [30]. Internet-based intervention has been recognized as one of the most effective tools for IBS self-help [31].

However, the effectiveness of internet-based self-help interventions for IBS, particularly in improving symptoms and mental health, remains insufficiently assessed. Existing meta-analyses on self-help interventions include both face-to-face and internet formats, with few studies and limited reliability [32]. Though recent studies have increased, debates persist regarding impacts on symptom severity [28,33], quality of life [34,35], depression [29,34], and anxiety [34,36]. Additionally, small sample sizes [26,27] and short durations (eg, 3 wk) [34] restrict generalizability. To date, the overall effectiveness of internet-based self-help interventions on IBS symptoms, psychological status, and quality of life remains unquantified. Therefore, this systematic review aims to quantitatively assess the clinical outcomes of internet-based self-help interventions compared with control groups (active control, usual care, or waitlist) in improving symptoms, psychological status, and quality of life in individuals with IBS, and to explore the effects of guidance format, intervention content, and duration on efficacy, providing evidence-based support for clinical practice.

## Methods

### Overview

This protocol for systematic review and meta-analysis adheres to the PRISMA (Preferred Reporting Items for Systematic Reviews and Meta-Analyses) guidelines (). The protocol was registered subsequent to determining methodological details via existing literature evaluation, and prior to the initiation of data extraction. During the later stages of data analysis, we made 3 methodological adjustments to the preregistered protocol to enhance methodological rigor and clinical interpretability. The language restrictions in the search strategy were removed to expand the evidence pool; the effect model selection approach was revised based on a priori conceptual hypotheses of between-study heterogeneity instead of I² statistics and Q-test *P* values; and the GRADE (Grading of Recommendations Assessment, Development and Evaluation) framework was incorporated for systematic evidence quality assessment. All adjustments only optimized the analysis method and did not change the core content of the original plan.

### Search Strategy

This systematic review conducted the literature search in accordance with the PRISMA-S (Preferred Reporting Items for Systematic Reviews and Meta-Analyses–search extension) guidelines [37]. A comprehensive search was performed across the following databases from their respective inception dates to June 25, 2025: PubMed (National Center for Biotechnology Information), Embase (Embase.com), Web of Science (Clarivate Analytics), Cochrane Library (Wiley), PsycINFO (Ovid), CINAHL (EBSCOhost), WANGFANG (Wanfang Data), CNKI (China National Knowledge Infrastructure), VIP (VIP Information), and CBM (China Biology Medicine Disc; CBMdisc Platform). A supplementary search was then implemented on March 9, 2026, after optimizing the search strategy. Notably, no multiple databases were accessed via the same platform during the search process. The search was executed without language or other restrictions, and no relevant filters were applied. No intentional search was conducted on trial registries, online resources, or print publications; nor were any alternative information sources or search methods used. The search strategy for this systematic review was developed based on approaches reported in prior studies [3,38], though it was not subjected to peer review. To identify additional potential studies, the reference lists of previous meta-analyses and included articles were manually screened. We will contact corresponding authors, domain experts, and manufacturers to obtain supplementary relevant information. Detailed search strategies for each database are presented in Multimedia Appendix 1.

### Inclusion and Exclusion Criteria

Inclusion criteria were as follows: (1) participants aged older than 16 years with IBS diagnosed by ROME criteria or professional medical personnel; (2) internet-delivered self-help interventions, including pure self-help interventions without therapist contact and guided interventions; (3) including active controls (placebo or other treatments) and inactive controls (routine treatment, waiting list); (4) outcomes include IBS symptom severity, quality of life, visceral sensitivity, anxiety, and depression, with IBS symptom severity and quality of life as primary outcomes, and others as secondary; (5) only include peer-reviewed randomized controlled trials (RCTs); and (6) no restrictions on language or publication year.

Exclusion criteria were as follows: (1) unavailable full texts; (2) reviews, conference abstracts, study protocols, and non-peer-reviewed studies; and (3) duplicate data or data that cannot be converted or obtained.

### Study Selection and Data Extraction

Initially, EndNote’s duplicate detection tool was used to identify overlapping references, which were subsequently removed following manual verification. Subsequently, 2 investigators (XX and TL) independently screened the titles and abstracts of the remaining references against the predefined inclusion and exclusion criteria, followed by full-text assessments to determine study eligibility. Any discrepancies arising during the screening or assessment process were resolved through discussion with a third investigator (FW). Data extraction will be double-checked, encompassing the following information: first author and year of publication; sociodemographic characteristics of the included studies (country, age, proportion of female participants, and sample size); diagnostic criteria for IBS; study location; intervention details (intervention content, intervention duration, and follow-up duration); control group conditions; and outcome measures.

### Methodological Quality Assessment

Two reviewers (XX and TL) assessed methodological quality using the Cochrane Risk of Bias 2.0, with discrepancies resolved by a third reviewer. The tool evaluates 5 key biases: randomization process, deviations from intended interventions, missing outcome data, measurement of the outcome, and selection of the reported result, each rated as “low risk,” “high risk,” or “some concerns.”

### Statistical Analysis

This systematic review pooled effect sizes using the standardized mean difference (SMD) based on the mean values and SDs of outcome measures in the intervention and control groups, in accordance with methods recommended in the Cochrane Handbook. Given the inconsistent outcome measurement tools across studies, SMD with 95% CIs was used as the effect size metric. Hedges *g*, a small-sample bias-corrected SMD, was the actual effect size calculated in this study, with interpretation criteria analogous to Cohen *d* (0.2=small effect, 0.5=moderate effect, 0.8=large effect) [39]. Results of this systematic review were interpreted as follows: inverse scores indicated improvements in IBS symptom severity, visceral sensitivity, and psychological status; positive scores reflected better quality of life.

Quantitative analyses for this systematic review were performed using R (version 4.5.0; R Core Team) software in the RStudio environment, with core analyses conducted using the following R packages: tidyverse, meta, and dmetar. Meta-analyses were carried out in accordance with the Hartung-Knapp-Sidik-Jonkman method recommended by the Cochrane Collaboration. Compared with the standard DerSimonian-Laird method [40], the Hartung-Knapp-Sidik-Jonkman method was preferred for its ability to estimate the weighted pooled mean with higher precision while reducing the false positive rate. Model selection was based on theoretical assumptions regarding the distribution of true effects, rather than statistical considerations [41,42]. Given the variability in participant characteristics and study protocols across included studies, a random-effects model was used for all analyses. Heterogeneity in this systematic review was quantified using the Q-test, I² statistic, and tau-squared (τ²). The I² statistic serves only as a relative quantitative measure of heterogeneity and does not directly reflect the magnitude of variability in true effects across different populations or settings, thus having limited clinical utility [43]. Thus, this study also reports the prediction interval (PI) to quantify the real-world implications of heterogeneity, providing a more clinically relevant range of effects for clinical practice [43].

To ensure the statistical power of subgroup analyses and meta-regression, only results with significant heterogeneity from meta-analyses comprising 10 or more studies were analyzed [44,45]. Subgroup analyses of postintervention data were performed based on prespecified intervention criteria to assess differences in effect sizes across subgroups. Categorical variables included the mode of health care delivery, control group type, intervention duration, and measuring tool. Meta-regression analyses were conducted for the primary outcome, with age and proportion of female participants as moderator variables. Meta-regression analyses were based on the random-effects framework to account for residual heterogeneity not explained by moderators. Subgroup analyses and meta-regression are inherently observational analytical approaches. Even when leveraging data from RCTs, they cannot establish causal relationships; instead, their core purpose is to explore potential moderator variables. The final reported outcomes in this review were fully consistent with the prespecified outcomes. In addition, sensitivity analyses were performed by sequentially excluding each individual study and recomputing the pooled effect size to assess the robustness of the results, thereby identifying studies with a substantial impact on the pooled effect. Visual inspection via funnel plots and Egger’s linear regression test was used to evaluate small-study effects [46]. Per methodological guidelines, reliable interpretation of funnel plot asymmetry requires at least 10 included studies; otherwise, the reliability of inferences is limited [47]. Meanwhile, when the number of studies is small, the statistical power of Egger’s test is significantly reduced, which may increase the risk of false-negative results [48]. If it is confirmed that there is a small research effect, then the trim-and-fill method will be used to correct the combined effect size. Statistical significance was set at *P*<.050.

### Evaluation of Evidence Quality

To assess the certainty of evidence, we performed a GRADE evidence quality assessment for the outcomes (ie, IBS symptom severity, quality of life, visceral sensitivity, depressive symptoms, and anxiety) based on the findings of this systematic review, in accordance with guidelines developed by the GRADE Working Group. Two reviewers (XX and TL) independently evaluated the risks of bias, inconsistency, indirectness, imprecision, and publication bias. Any discrepancies were resolved through discussion with a third reviewer (FW) to reach a consensus.

## Results

### Search Results

**Figure 1.**
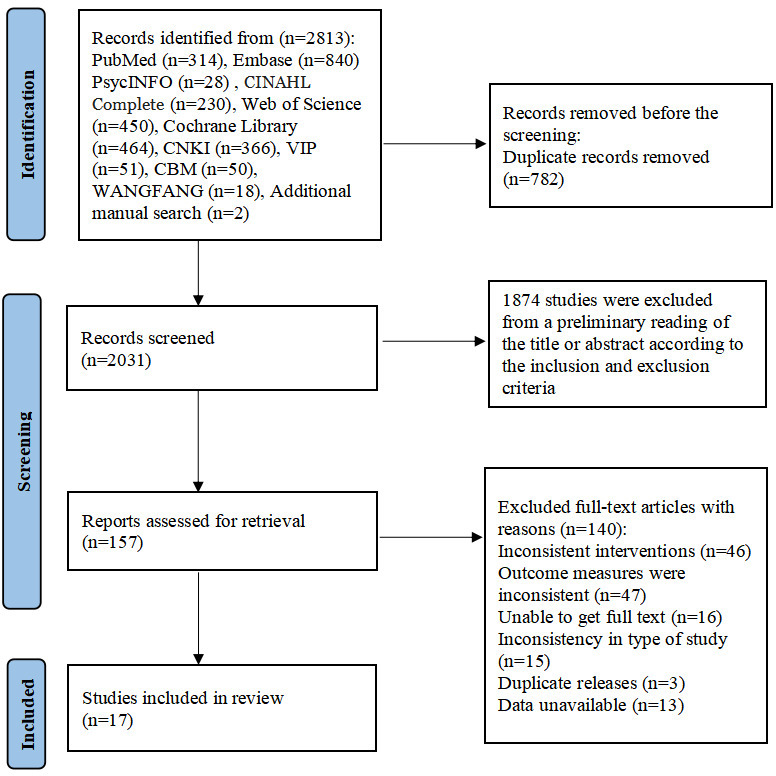
PRISMA (Preferred Reporting Items for Systematic Reviews and Meta-Analyses) flow diagram of the literature search and study selection.

The study selection process is shown in Figure 1. Initial database searches yielded 2813 studies. After removing 782 duplicates, 2031 remained. Title and abstract screening based on inclusion criteria led to full-text evaluation of 1874 studies, with 140 excluded. Ultimately, 17 studies were incorporated into the meta-analysis [26-30,33-36,49-56].

### Characteristics of Included Studies

The basic characteristics of the included studies are summarized in Multimedia Appendix 2 [26-30,33-36,49-56]. A total of 2289 participants were enrolled, with a mean age ranging from 18.53 to 45.4 years and a female predominance (56.7% to 100%). For the diagnosis of IBS, the criteria adopted were primarily Rome III (n=10) and Rome IV (n=6), with 1 study using the Rome II criteria. The majority of studies were conducted in North America (n=7), followed by Europe (n=6), the Asia-Pacific region (n=3), and the Middle East (n=1). More than half of the included studies (n=9) were single-center trials, while 8 were multicenter trials.

All interventions were delivered via the Internet. The predominant intervention was CBT (n=11); the remaining interventions included yoga (n=2), progressive muscle relaxation (n=1), eHealth self-management (n=1), gut-directed hypnotherapy combined with CBT components (n=1), and online education (n=1). Most studies (n=15) adopted a guided delivery mode. For the control groups, 9 were inactive controls (usual care or waitlist), whereas the remaining 8 were active controls encompassing placebo, written expression, face-to-face education, psychoeducation, active control app, Internet-delivered stress management, CBT without exposure, and probiotics alone. The intervention lasted 3 to 10 weeks. Most studies (n=12) included a follow-up period, but only 8 provided complete follow-up data.

### Quality Assessment

**Figure 2.**
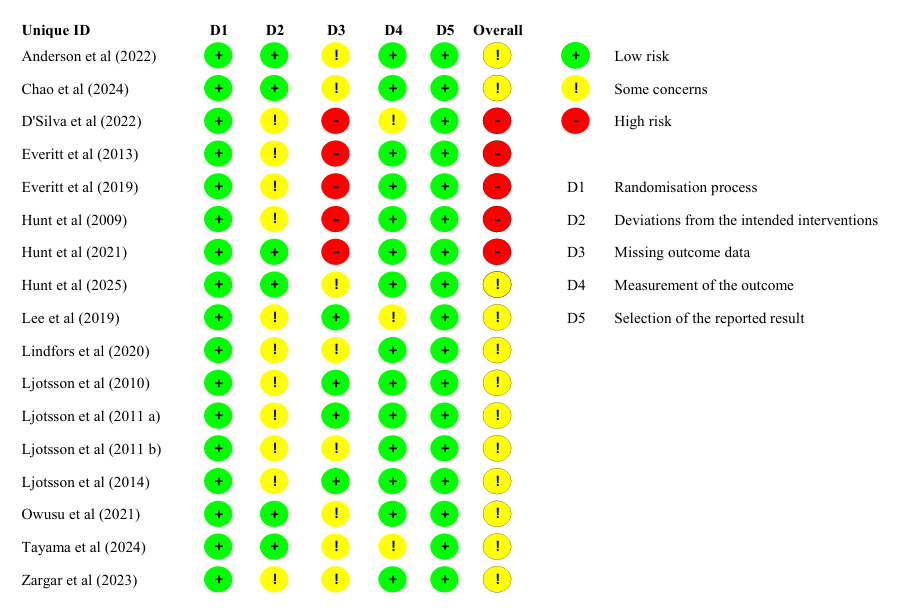
ROB 2.0 traffic light plot of included randomized controlled trials [26-30,33-36,49-56].

Risk of bias assessments are presented in Figure 2. Among these RCTs, 12 studies (70.6%) were judged to have some concerns, while 5 studies (29.4%) were assessed as having a high risk of bias.

### Effectiveness of Internet-Based Self-Help Interventions

#### Severity of IBS Symptoms

**Figure 3.**
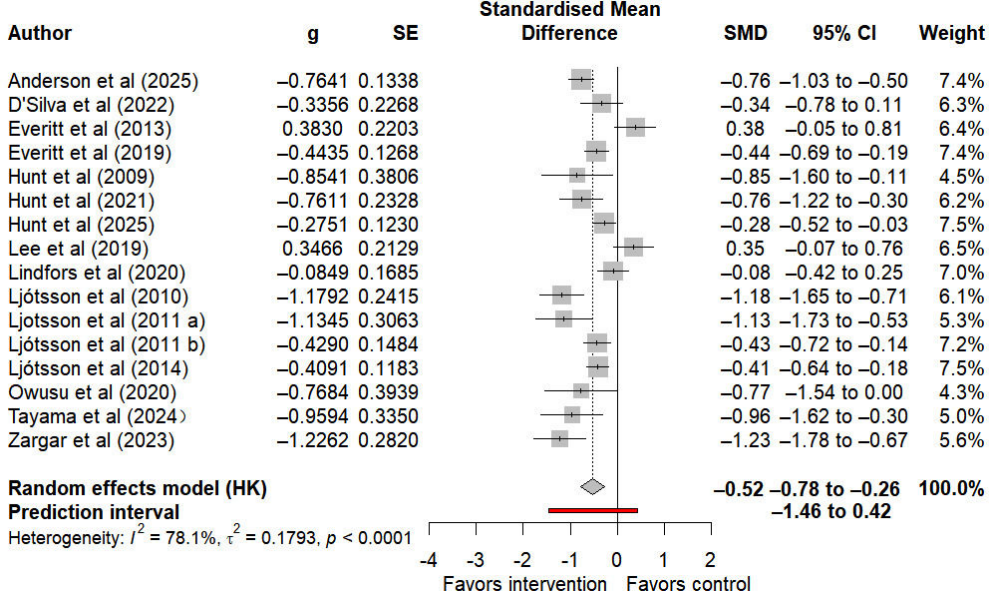
Forest plot of the effect on the severity of irritable bowel syndrome symptoms after internet-based self-help interventions [26-30,33-36,49-54,56].

16 studies assessed IBS symptom severity, of which 8 provided follow-up data. Compared with control groups, internet-based self-help interventions were associated with a moderate, statistically significant reduction in postintervention IBS symptom severity (SMD −0.52, 95% CI −0.78 to −0.26, 95% PI −1.46 to 0.42; I²=78.1%, τ²=0.1793, *P*<.001) (Figure 3). Note that the 95% CI reflects the statistical certainty around this pooled average effect. While the wide 95% PI, which spanned both clinically meaningful benefit and potential harm, reflects substantial variability across populations and clinical settings, indicating both meaningful benefit and no/little effect in future studies. Given the high heterogeneity of this outcome and the low certainty of evidence according to the GRADE assessment (Table 1), the generalizability of this average effect should be viewed with caution. In addition, no statistically significant effect was observed at follow-up (SMD −0.35, 95% CI −0.80 to 0.10, 95% PI −1.52 to 0.82; I²=77.3%, τ²=0.2115, *P*<.001) (Figure S1 in Multimedia Appendix 3 [26-30,33-36,49-56]).

**Table 1.** GRADE (Grading of Recommendations Assessment, Development and Evaluation) evidence quality assessment.

Outcome indicators	Certainty assessment							Number of patients		Effect, absolute (95% CI)	Certainty	Importance
	Number of studies	Study design	Risk of bias	Inconsistency	Indirectness	Imprecision	Other considerations	Intervention	Comparison			
Severity of IBS^a^ symptoms	16	Randomized trials	Serious^b^	Serious^c^	Not serious	Not serious	None	1006	1002	SMD^d^ 0.52 SD lower (95% CI −0.78 to −0.26)	⨁⨁◯◯ Low^b^^c^	CRITICAL
Quality of life	13	Randomized trials	Serious^b^	Serious^c^	Not serious	Not serious	Publication bias strongly suspected^e^	793	804	SMD 0.58 SD higher (95% CI 0.23 to 0.90)	⨁◯◯◯ Very low^e^	CRITICAL
Visceral sensitivity	8	Randomized trials	Not serious	Serious^c^	Not serious	Not serious	Publication bias strongly suspected^e^	572	580	SMD 0.55 SD lower (95% CI −0.89 to −0.21)	⨁⨁◯◯ Low^e^	CRITICAL
Depressive symptoms	9	Randomized trials	Serious^b^	Not serious	Not serious	Not serious	None	665	680	SMD 0.14 SD lower (95% CI −0.27 to −0.01)	⨁⨁⨁◯ Moderate^b^	IMPORTANT
Anxiety	7	Randomized trials	Not serious	Not serious	Not serious	Serious^f^	Publication bias strongly suspected^e^	560	552	SMD 0.03 SD lower (95% CI −0.34 to 0.27)	⨁⨁◯◯ Low^f^	IMPORTANT

a IBS: irritable bowel syndrome.

b Most studies did not explicitly report blinding of participants, researchers, and outcome assessors, and the majority of included trials were judged to have unclear or high risk of bias. All outcome measures relied on subjective scales, which may have overestimated treatment effects. Consequently, the certainty of the evidence was downgraded by one level due to concerns about the risk of bias.

c A wider prediction interval indicates a larger potential variability in effect values, encompassing both clinically significant benefits and the possibility of no effect or even potential harm. Significant heterogeneity between studies reflects notable differences in effect estimates across individual studies. Therefore, based on the assessment criteria for inconsistency of evidence, this systematic review has downgraded the certainty of the evidence by one level.

d SMD: standardized mean difference.

e The Egger intercept for quality of life was significant (*P*=.02), using Duval and Tweedie’s trim and fill method. The results added 4 studies, but the effect of the intervention still did not change.

f The 95% CI is wide and crosses the line of no effect, suggesting uncertainty in the true effect estimate. Therefore, the certainty of evidence was downgraded by one level for imprecision.

We explored sources of heterogeneity in postintervention outcomes using subgroup analyses and meta-regression. Subgroup analysis findings (Table 2 and Figures S2-S6 in Multimedia Appendix 3 [26-30,33-36,49-56]) showed that between-group differences for control group type (*P*=.78), intervention content (*P*=.49), intervention duration (*P*=.30), and the type of measuring tool (*P*=.051) were not statistically significant, suggesting these factors were not primary sources of heterogeneity. In contrast, health care contact (*P*=.04) emerged as one of the key contributors to high heterogeneity. Meta-regression analyses were also conducted to examine the association between study characteristics (age and female ratio) and heterogeneity (Table 3; Figures S7 and S8 in Multimedia Appendix 3 [26-30,33-36,49-56]). However, neither age (*P*=.77) nor female ratio (*P*=.27) was statistically significant, indicating they were not major sources of heterogeneity.

**Table 2.** Subgroup analysis results of internet-based self-help interventions on irritable bowel syndrome (IBS) symptom severity and quality of life.

Outcome indicator and moderate variable	SMD^a^ (95% CI)	*I*² (%)	*P* value
IBS symptom severity
Control group type			.78
Active control	−0.48 (−0.82 to −0.15)	70.2	.003
Inactive control	−0.55 (−1.02 to −0.09)	83.4	<.001
Intervention content			.49
CBT^b^	−0.47 (−0.81 to −0.12)	78.7	<.001
Other	−0.64 (−1.21 to −0.07)	78	.001
Intervention duration			.30
≤6 weeks	−0.33 (−1.03 to 0.37)	88.9	<.001
>6 weeks	−0.63 (−0.87 to −0.39)	56.5	.01
Health care contact			.04
Guided	−0.49 (−0.78 to −0.20)	80.2	<.001
Self-help interventions	−0.76 (−0.81 to −0.72)	0	.99
Measuring tool			.051
IBS-SSS^c^	−0.30 (−0.71 to 0.12)	81.9	<.001
GSRS-IBS^d^	−0.73 (−1.06 to −0.41)	71.7	<.001
Quality of life
Control group type			.14
Active control	0.38 (−0.13 to 0.89)	74.7	<.001
Inactive control	0.79 (0.31 to 1.26)	69.2	.006
Intervention content			.85
CBT	0.55 (0.19 to 0.92)	74.6	<.001
Other	0.63 (−0.37 to 1.62)	83.4	<.001
Intervention duration			.87
≤6 weeks	0.54 (−0.50 to 1.57)	84.3	<.001
>6 weeks	0.60 (0.26 to 0.94)	73	<.001

a SMD: standardized mean difference.

b CBT: cognitive behavioral therapy.

c IBS-SSS: Irritable Bowel Syndrome Symptom Severity Scale.

d GSRS-IBS: Gastrointestinal Symptom Rating Scale-Irritable Bowel Syndrome.

**Table 3.** Meta-regression results of internet-based self-help interventions on irritable bowel syndrome (IBS) symptom severity and quality of life.

Outcome indicator and covariate	β coefficient (SE)	95% CI	*T* test	*P* value
IBS symptom severity				
Age (years)	0.01 (0.02)	−0.03 to 0.04	0.29	.77
Female ratio	0.01 (0.01)	−0.01 to 0.04	1.16	.27
Quality of life				
Age (years)	−0.04 (0.02)	−0.10 to 0.01	−1.85	.09
Female ratio	−0.00 (0.02)	−0.05 to 0.04	−0.15	.88

#### Quality of Life

**Figure 4.**
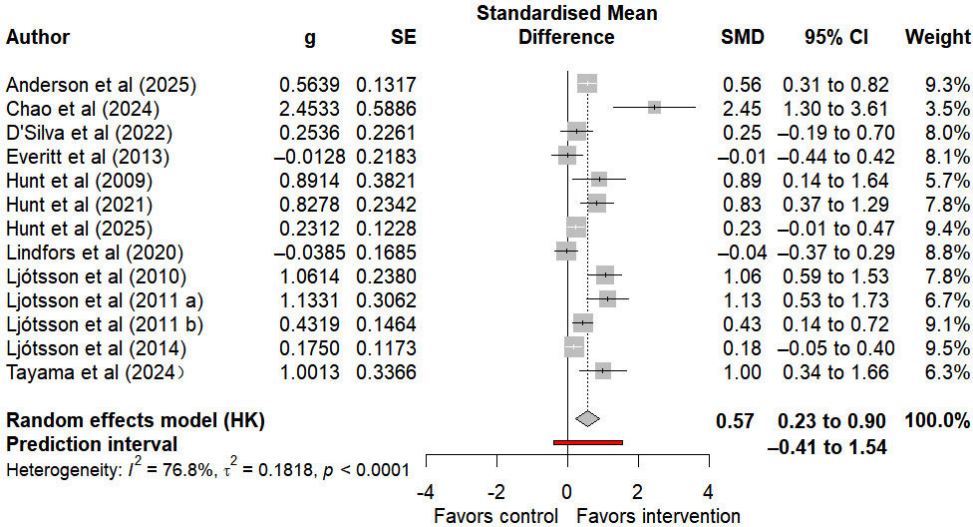
Forest plot of the impact of internet-based self-intervention on quality of life in irritable bowel syndrome [27-30,34-36,49,50,52,53,55,56].

13 studies assessed quality of life in individuals with IBS, of which 6 provided follow-up data. Compared with control groups, internet-based self-help interventions were associated with a moderate, statistically significant improvement in postintervention QoL (SMD 0.57, 95% CI 0.23 to 0.90, 95% PI −0.41 to 1.54; I²=76.8%, τ²=0.18, *P*<.001) (Figure 4). Here, the 95% CI denotes the statistical certainty of this average improvement effect, while the 95% PI spanned both beneficial and null effects, indicating substantial variability in intervention outcomes across different populations, clinical settings, and implementation strategies. Given the high heterogeneity of this outcome and the very low certainty of evidence according to GRADE (Multimedia Appendix 3 [26-30,33-36,49-56]), the credibility and applicability of this conclusion should be assessed with caution. Furthermore, no statistically significant effect was observed at follow-up (SMD 0.12, 95% CI −0.02 to 0.25, 95% PI −0.07 to 0.31; I²=0%, τ²=0, *P*=.80) (Figure S9 in Multimedia Appendix 3 [26-30,33-36,49-56]).

Subgroup analyses of postintervention data (Table 2 and Figures S10-S12 in Multimedia Appendix 3 [26-30,33-36,49-56]) revealed that the control group type (*P*=.14), intervention content (*P*=.85), and intervention duration (*P*=.87) were not the key influencing factors for high heterogeneity. Due to the limited number of subgroup studies, health care contact was not included as a moderating variable. Meta-regression analyses (Table 3 and Figures S13 and S14 in Multimedia Appendix 3 [26-30,33-36,49-56]) demonstrated that neither age (*P*=.09) nor the female ratio (*P*=.88) reached statistical significance, indicating they were not major sources of heterogeneity.

#### Visceral Sensitivity

**Figure 5.**
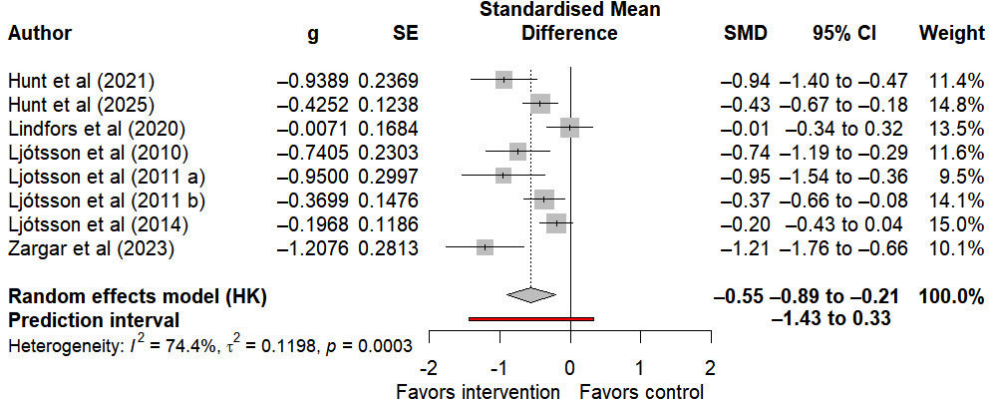
Forest plot of the effect of internet-based self-intervention on visceral sensitivity in irritable bowel syndrome [29,30,34,35,49,52-54].

The visceral sensitivity was used to assess gastrointestinal symptom-specific anxiety in individuals with IBS. A total of 8 studies demonstrated that internet-based self-help interventions yielded a statistically significant improvement in visceral sensitivity among these patients, with a moderate to large effect size (SMD −0.55, 95% CI −0.89 to −0.21, 95% PI −1.43 to 0.33; I²=74.4%, τ²=0.1198, *P*<.001) (Figure 5). Notably, the wide span of the 95% PI reflects the uncertainty surrounding the magnitude of intervention effects in future studies. The certainty of the GRADE evidence for this outcome is low, and there is high heterogeneity (Multimedia Appendix 3 [26-30,33-36,49-56]), suggesting that the effect of this intervention on the improvement of visceral sensitivity varies considerably across different studies, and the conclusions need to be interpreted in the context of specific scenarios.

#### Depressive Symptoms

**Figure 6.**
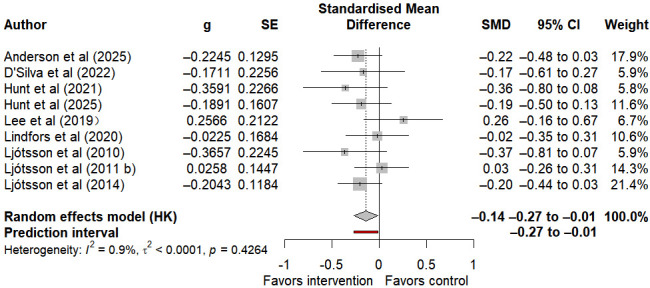
Forest plot of internet-based self-help interventions for depression in irritable bowel syndrome [29,30,34,36,49-53].

9 studies reported on depressive symptoms in individuals with IBS. Compared with control groups, internet-based self-help interventions were associated with statistically significant improvement in depressive symptoms (SMD −0.14, 95% CI −0.27 to −0.01, 95% PI −0.27 to −0.01; I²=0.9%, τ²<0.001, *P*=.43) (Figure 6). The certainty of the evidence according to GRADE is moderate (Multimedia Appendix 3 [26-30,33-36,49-56]). Currently, it is possible to confirm the impact of internet-based self-help interventions on depressive symptoms in individuals with IBS.

#### Anxiety

**Figure 7.**
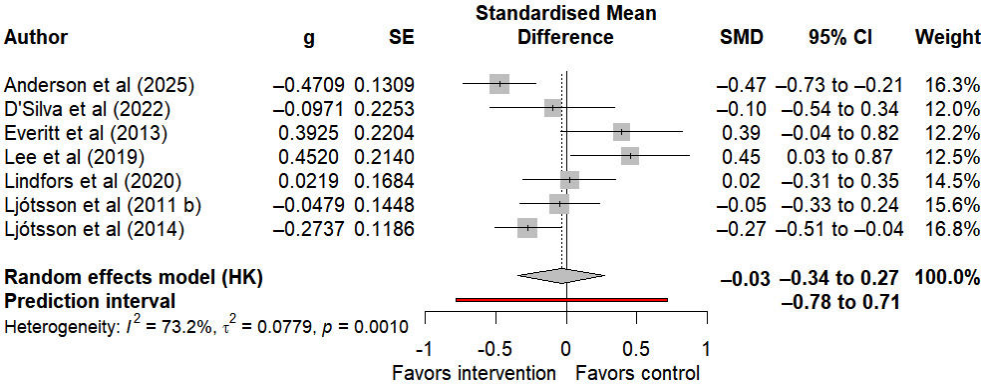
Forest plot of internet-based self-help interventions for anxiety in irritable bowel syndrome [28,34,36,49-52].

7 studies reported on anxiety symptoms in individuals with IBS. Meta-analysis results indicated that internet-based self-help interventions were not associated with a statistically significant effect on anxiety (SMD −0.03, 95% CI −0.34 to 0.27, 95% PI −0.78 to 0.71; I²=73.2%, τ²=0.08, *P*=.001) (Figure 7). Considering the low certainty of evidence for this outcome (Multimedia Appendix 3 [26-30,33-36,49-56]), current evidence is insufficient to support the effectiveness of internet-based self-help interventions in improving anxiety symptoms in individuals with IBS.

### Sensitivity Analysis

A leave-one-out sensitivity analysis was conducted on postintervention results for all outcome measures to verify the robustness of pooled estimates across different outcomes after excluding each individual study. No substantial changes were observed in the pooled effect sizes or their corresponding directions in this systematic review following the exclusion of any single study, thus confirming the good overall robustness of the results (Figures S15-S19 in Multimedia Appendix 3 [26-30,33-36,49-56]).

### Small-Study Effects

Small-study effects were assessed using funnel plots and Egger’s test. Funnel plots were generated for the outcomes with more than 10 included studies, namely IBS symptom severity (Figure S20 in Multimedia Appendix 3 [26-30,33-36,49-56]) and quality of life (Figure S21 in Multimedia Appendix 3 [26-30,33-36,49-56]). These funnel plots exhibited asymmetry, which may indicate potential publication bias, as well as reflect heterogeneity and random chance across studies. Egger’s regression intercept was applied to all outcomes to evaluate bias. The nonsignificant Egger’s intercepts for IBS symptom severity (*P*=.27) and depressive symptoms (*P*=.90) suggested the absence of small-study effects. However, Egger’s intercept for quality of life (*P*=.01), visceral sensitivity (*P*=.02), and anxiety (*P*=.03) was statistically significant, prompting the application of the Duval and Tweedie trim-and-fill method. For quality of life, 4 additional studies were included (SMD 0.33, 95% CI −0.08 to 0.75); while Visceral Sensitivity added 3 studies (SMD −0.32, 95% CI −0.71 to 0.06). The results of the meta-analysis indicated that the intervention effects of these 2 outcomes significantly changed after the supplementary studies were added, suggesting that the robustness of the relevant evidence was poor. When interpreting the conclusions, caution is required. For anxiety, after adding 2 supplementary studies, the combined effect did not significantly change (SMD −0.20, 95% CI −0.55 to 0.16), suggesting that the influence of small studies on the combined results of this outcome was relatively small.

### GRADE Assessment

The GRADE evidence certainty assessment indicated that most outcomes were rated as having very low to moderate certainty (Table 1). Most studies did not explicitly report blinding procedures for participants, interventionists, and outcome assessors, and all outcomes were subjective, undermining the reliability, so we downgraded the evidence certainty for severity of IBS symptoms, quality of life, and depressive symptoms by one level due to risk of bias. Furthermore, substantial heterogeneity in IBS symptom severity, quality of life, and visceral sensitivity contributed to variability in effect estimates. Additionally, the 95% CIs and PIs were wide, encompassing not only clinically meaningful benefits but also no effect or potential harm. We therefore further downgraded evidence certainty for IBS symptom severity, quality of life, and visceral sensitivity by one level due to inconsistency. For quality of life, visceral sensitivity, and anxiety outcomes, Egger’s test confirmed small-study effects. Although the trim-and-fill adjustment did not alter the intervention effect, it still indicated potential bias, prompting an additional one-level downgrade for this outcome. For anxiety outcomes, the 95% CIs were wide and crossed the null effect line, signaling uncertainty in estimating the true effect. We thus downgraded evidence certainty by one level due to imprecision. Overall, GRADE evidence certainty findings substantially impact the credibility of the pooled effect estimates.

## Discussion

### Principal Findings

This study extends beyond prior similar reviews focused on internet-delivered CBT by expanding the scope of intervention modalities evaluated and quantifying the effects of key implementation factors, including intervention duration, guidance mode, and core intervention content, on treatment efficacy, thereby providing more actionable evidence for clinical practice [3]. Quantitative synthesis revealed that internet-based self-help interventions led to significant postintervention improvements in IBS symptom severity, quality of life, visceral sensitivity, and depressive symptoms for individuals with IBS, with small-to-moderate effect sizes. These short-term benefits, however, were not sustained at follow-up, reflecting the limited durability of the intervention’s long-term effects. Notably, no significant improvements in anxiety symptoms were observed with these interventions, and high heterogeneity was present across all primary outcomes. Given that GRADE assessments rated the certainty of evidence as very low to moderate, these findings must be interpreted cautiously in the context of the study’s methodological limitations.

### Symptom Severity

IBS symptom severity serves as a primary outcome measure for evaluating the efficacy of internet-based self-help interventions. Quantitative findings demonstrated that these interventions yielded significant short-term improvements in IBS symptom severity with superior efficacy relative to control conditions, which is consistent with the conclusions of previous similar studies and further validates the value of internet-based self-help interventions in the symptom management of IBS [3]. However, the 95% PIs had a wide range, indicating that intervention effects may exhibit substantial variability across different clinical settings. Individuals with IBS who are proficient in internet use and have high health care accessibility can better complete structured intervention modules and achieve more pronounced symptom improvements; whereas those in remote areas or with low digital health literacy may experience reduced adherence due to operational difficulties and lack of guidance, yielding minimal or even no significant benefits from the interventions.

Subgroup analyses further indicate that variations in health care contact constitute a key source of high heterogeneity. We found that guided self-help interventions may yield greater benefits, which is consistent with the findings of a meta-analysis indicating that increased therapist contact can enhance the efficacy of self-help interventions [57]. This may be attributed to the fact that low-intensity guidance from health care professionals enhances intrinsic motivation and treatment adherence among individuals with IBS and fosters positive therapeutic relationships, thereby sustaining the improvements in their quality of life [28,58]. Different scales have fundamental differences in the construction definition and evaluation dimensions of symptom severity, which may have certain impacts on the presentation of effect values. However, the subgroup analysis results show that this scale difference is not the main source of heterogeneity in this study. Specifically, studies using the Gastrointestinal Symptom Rating Scale-Irritable Bowel Syndrome as the assessment tool reported larger effect sizes. This scale is a patient-reported outcome measure that focuses on the daily fluctuations of core IBS symptoms and can more sensitively capture subtle improvements in individuals’ subjective perceptions [59]. In contrast, the effect value of the Irritable Bowel Syndrome-Symptom Severity Scale is relatively small and lacks statistical significance. As an assessment tool commonly used in clinical practice, the Irritable Bowel Syndrome-Symptom Severity Scale evaluation criteria place more emphasis on objective symptom indicators and may, to some extent, overlook the changes in individual subjective perception [60,61]. Meta-regression analyses demonstrated that neither mean age nor the proportion of female individuals exerted a significant effect on effect sizes, ruling out the possibility that demographic characteristics constitute a primary source of heterogeneity.

Due to the risk of bias, high heterogeneity stemming from health care contact variability, and wide 95% PIs, the GRADE certainty of evidence for this outcome was rated as low, which was downgraded for risk of bias and insufficient consistency. This low certainty of evidence does not negate the inherent value of these interventions, but rather reflects a gap between the current evidence and real-world practice. This indicates that while internet-based self-help interventions have a well-established short-term beneficial effect on IBS symptom severity, caution is warranted when generalizing this conclusion. In real-world implementation, a comprehensive consideration of factors including intervention plan, digital health literacy among individuals with IBS, and local health care service capacity is required to maximize intervention efficacy and enhance the credibility of evidence.

### Quality of Life

Compared with the general population, individuals with IBS experience a significant impairment in their quality of life [62]. Thus, improving quality of life is one of the core objectives of self-management for IBS. Meta-analysis findings demonstrated that internet-based self-help interventions produced a moderate short-term improvement in the quality of life among individuals with IBS, which is consistent with the conclusions of a systematic review focusing on digital health self-management for IBS [63], providing more robust evidence to support improvements in quality of life among individuals with IBS. However, wide 95% PIs indicate that these beneficial effects exhibit substantial variability across different clinical settings, which necessitates a comprehensive evaluation based on specific implementation conditions.

Given the high heterogeneity of the results, subgroup analyses were conducted based on potential moderating variables such as the type of control group, intervention period, and intervention content. It was found that none of these factors was the main source of heterogeneity. This result indicates that although there are differences in the types of controls, intervention periods, and intervention contents among different studies, these differences did not significantly affect the overall heterogeneity among the studies. The subgroup results showed that, compared with the active control group, the effect size in the inactive control group was higher. Inactive control groups lack targeted intervention resources, resulting in a larger efficacy gap relative to internet-based self-help interventions and thus making the value of these interventions more prominent. Second, interventions with a duration of more than 6 weeks yielded superior improvements in quality of life, which aligns with the conclusion by Liegl et al [32] that self-help interventions require sufficient time to consolidate behavioral changes. Melchior et al [64] noted that quality of life in individuals with IBS is jointly influenced by multiple factors, including gastrointestinal symptoms, psychological distress, and coping strategies, and improvements in these factors depend on the long-term consolidation of health behaviors such as dietary modifications and stress management. Extending the intervention duration enables individuals with IBS to engage in repeated practice of relaxation training and trigger identification skills within online modules, which facilitates the gradual reduction of excessive attention to gastrointestinal symptoms and thus results in a sustained improvement in quality of life. The subgroup analysis results of this study confirmed the aforementioned theoretical expectations.

However, the subgroup interaction test results of this study showed that the differences in effect sizes of these factors did not reach a statistically significant level, and thus were not identified as the main source of heterogeneity. This might be due to sample size limitations or other unidentified confounding factors resulting in insufficient statistical test power, preventing the detection of the significant contributions of these factors to heterogeneity. This suggests that the improvement in the quality of life of IBS patients may be more dependent on the core attributes of internet self-help interventions, such as convenience, autonomy, and targeted content provision, rather than the differences in specific implementation conditions, or the influence of these implementation conditions may be masked by other unidentified factors. Meta-regression analyses demonstrated that mean age and the proportion of female individuals were not a source of heterogeneity.

Due to the presence of bias risks in most studies, high unexplained heterogeneity, and a wide 95% CI range, the GRADE certainty of the evidence was rated as very low; moreover, the existence of small sample effects further downgraded the quality of the evidence, making the effect estimates highly susceptible to potential biases.

Based on these findings, in future research, when designing Internet-based self-help intervention programs for IBS, one can flexibly choose the core content of the intervention, reasonably plan the intervention period, and set the type of control according to the research purpose. More importantly, it is necessary to focus on exploring other potential sources of heterogeneity while increasing the sample size to reduce the bias of small samples, and further optimize the targeted and stable nature of the intervention program, providing more solid evidence to support the improvement of the long-term quality of life of IBS patients.

### Visceral Sensitivity

Visceral sensitivity centers on gastrointestinal symptom-specific anxiety and denotes the cognitive, emotional, and behavioral responses triggered by fear of gastrointestinal symptoms. It is a key factor in the disease progression of IBS and also a well-established therapeutic target [57]. This systematic review found that internet-based self-help interventions exert a large short-term effect on visceral hypersensitivity, which is consistent with the biopsychosocial model of brain-gut axis dysfunction in IBS [58]. As Wolitzky-Taylor et al [65] note, anxiety toward visceral sensations in individuals with IBS triggers avoidant behaviors and hypervigilance. In contrast, through structured learning within internet-based self-help interventions, these individuals can adopt strategies including relaxation training and exposure exercises to reduce catastrophic symptom-related thinking, alleviate gastrointestinal hypersensitivity, and food-related fear, and thus achieve symptomatic improvements [30]. Furthermore, as a mediating variable, visceral hypersensitivity facilitates the indirect improvement of core IBS symptoms and quality of life among individuals with IBS [30,65].

It is important to note that wide 95% PIs indicate that this effect may exhibit substantial variability in future research, which warrants cautious interpretation in the context of clinical practice. The GRADE certainty of evidence for this outcome was rated as low. Its rating was downgraded because the high heterogeneity led to inconsistent effect estimation results and the presence of small study effects, which indicates that the significant short-term effect of this outcome still requires validation from additional homogeneous studies.

### Psychological Distress

Psychological distress, including anxiety and depression, constitutes a common symptom cluster among individuals with IBS [66]. Psychosocial moderators in IBS are widely emphasized, as psychological factors influence IBS symptoms, modify illness-related behaviors, and contribute to adverse health outcomes [67]. However, findings from this systematic review in the domain of psychological symptoms show differences from those of previous research [3,68], whereby internet-based self-help interventions statistically improve depression, but no significant effect has been observed in the improvement of anxiety.

Findings from the meta-analysis showed a slight but statistically significant improvement in the depressive symptoms of patients with IBS. The 95% PI was consistent with the CI, not crossing the zero-effect line, suggesting that the improvement effect of this intervention on depression was stable and could show consistent minor benefits in different research scenarios. At the same time, the heterogeneity of this outcome was extremely low, further confirming the consistency of the results among studies. This conclusion is different from the previous research conclusion that such interventions had no significant improvement effect on depression. The occurrence of this positive result may be due to the targeted improvement of IBS physical symptoms by the intervention, which led to indirect psychological benefits. Although the studies included were mainly aimed at improving core physical symptoms such as abdominal pain and bloating, the relief of physical symptoms effectively reduced the patients’ sense of disease burden caused by chronic diseases, thereby alleviating the associated depressive emotions [69]. In addition, most of the included studies provided psychological intervention modules, integrating relevant content of CBT, such as stress management, cognitive regulation, etc. These standardized modules may still exert a slight antidepressant effect, and the extremely low research heterogeneity also indicates that this type of internet self-help intervention has stability in improving depression. The evidence GRADE rating for the depression improvement effect of this study is moderate. It was slightly downgraded due to the certain risk of bias in the included studies, but this confirms that the conclusion has high credibility.

On the contrary, the self-help intervention based on the Internet has not shown statistically significant improvement in the anxiety symptoms of patients with IBS. This result is consistent with the conclusions of previous studies, and its evidence has a GRADE rating of low, indicating weak evidence strength. The difference in the intervention effect between depression and anxiety may stem from the fundamental differences in the pathogenesis and influencing factors of the two in the IBS population. The anxiety of patients with IBS is often directly related to visceral hypersensitivity and fear of symptom recurrence [70]. Such anxiety symptoms require more targeted and interactive professional psychological intervention, such as exposure therapy for symptom-related fears, one-on-one cognitive restructuring guidance, etc. However, the current low-intensity, noninteractive Internet self-help intervention model included in the study may not solve this specific type of anxiety problem. Additionally, there are differences in measurement tools, and the design characteristics of different questionnaires may directly affect the accuracy of results and effect estimation [71]. The sample size of the research on anxiety is limited, and the statistical test power is insufficient, which directly reduces the credibility of the conclusion. The results of the correlation analysis of anxiety symptoms have considerable inaccuracy, and the effect estimation is prone to being interfered with by potential biases, making it impossible to rule out the possibility of the existence of the real effect; at the same time, there is a small study effect for this outcome, and the certainty of the evidence is thus further reduced. Ultimately, it is rated as low.

For individuals with IBS, self-help interventions based on the Internet can not only improve the physical symptoms, but also exert a mild antidepressant effect. This is a cost-effective, comprehensive intervention method. However, such interventions have no clear effect on the improvement of anxiety symptoms. When clinicians apply Internet self-help interventions to alleviate the physical symptoms of individuals, they need to supplement more targeted psychological support measures for individuals with IBS who have co-occurring anxiety symptoms, so as to achieve comprehensive management of somatic and psychological symptoms in individuals with IBS.

### Implications for Clinical Practice

From the perspective of clinical practice, in response to the socioeconomic burden imposed by IBS, it is imperative to both establish the clinical effectiveness of intervention strategies and develop innovative service models directly accessible to individuals with IBS, thereby enhancing their access to appropriate care. Internet-based self-help interventions leverage digital platforms to transcend temporal and spatial constraints, thereby facilitating autonomous access to intervention resources for individuals with IBS. Through web-based delivery modes, these individuals can independently acquire basic IBS-related knowledge and coping skills [23], like dietary guidance, relaxation training, and symptom management [56].

This intervention model preserves the structured nature of interventions, enhances the flexibility and accessibility of participation for individuals with IBS, and reduces health care costs, making it particularly suitable for individuals with IBS who seek frequent medical care or those living in regions with limited health care resources. Despite uncertainties regarding long-term efficacy and the low quality of available evidence, internet-based self-help interventions still exhibit certain clinical value for symptom management in individuals with IBS and may therefore be considered a component of comprehensive symptom management plans for IBS.

### Limitations

This study has several limitations. First, in the literature search phase, we did not access relevant online resources or clinical trial registries and only included studies published in Chinese and English, potentially introducing selection and language bias. Second, regarding study design and outcome assessment, most included studies did not explicitly report blinding of participants, interventionists, and outcome assessors. Additionally, all outcome measures relied on self-reported scales, introducing subjective bias that may undermine the accurate assessment of the clinical efficacy of these internet-based self-help interventions.

Third, measurement tool heterogeneity was evident: the same outcome constructs were evaluated using different assessment scales. Although a methodologically sound SMD was applied for data pooling, different scales demonstrate inherent differences in the construct definitions, assessment dimensions, and scoring approaches of core outcomes. This can result in ambiguous construct validity, exacerbate measurement heterogeneity, and indirectly reduce the accuracy and interpretability of pooled effect sizes. Fourth, high heterogeneity existed across all included studies. Although we conducted subgroup analyses and meta-regression per prespecified criteria, unidentified potential moderators likely remain, leading to an incomplete characterization of heterogeneity sources. Additionally, some RCTs included for specific outcomes had small sample sizes, resulting in insufficient statistical power and an inability to accurately evaluate true intervention effects.

Given the aforementioned limitations, the findings of this study should be interpreted cautiously. Future research should conduct large-sample, multicenter RCTs and prioritize multidimensional outcome measures. Additionally, it is imperative to identify the core active components of standardized intervention protocols, including intervention duration, frequency, and guidance intensity, and harmonize assessment tools for core constructs. This will mitigate the impact of measurement heterogeneity on study outcomes and further validate the true efficacy of internet-based self-help interventions for individuals with IBS.

### Conclusions

This meta-analysis provides a comprehensive assessment of the latest evidence for internet-based self-help interventions in the management of individuals with IBS. Findings demonstrate these interventions produce significant short-term, small-to-moderate effect size improvements in disease-specific symptom severity, quality of life, visceral sensitivity, and depressive symptoms among this population, thus providing multi-outcome evidence to support their use as an adjunct to conventional care. It also broadens the scope of applicable intervention modalities, includes visceral sensitivity as a core outcome measure, and quantifies how key implementation factors influence intervention effectiveness, ultimately building a more comprehensive evidence base for digital interventions in the care of individuals with IBS. In real-world clinical practice, internet-based self-help interventions offer high accessibility, convenience, and cost-effectiveness, making them particularly suitable for individuals with IBS in resource-limited health care settings or those who seek frequent clinical care. They can act as a key component of comprehensive disease management plans and inform the development of intervention strategies for primary care and telemedicine platforms.

However, these findings are subject to substantial uncertainty. Notably, the short-term benefits of these interventions are not sustained at follow-up; no significant improvements in anxiety symptoms are observed among individuals with IBS, and high heterogeneity exists across primary outcomes. Additionally, GRADE evidence certainty was rated as very low to moderate for all outcomes; moderate risk of bias, alongside effect variability associated with wide 95% PIs, further weakens the credibility of the evidence base. This highlights the need for extreme caution when translating these findings into clinical practice. In clinical practice, the implementation of internet-based self-help interventions should consider individual factors (eg, digital health literacy) and contextual factors (eg, local health care service capacity) to optimize their clinical value in a targeted manner.

## Supplementary material

10.2196/87216 Multimedia Appendix 1 Search strategy.

10.2196/87216 Multimedia Appendix 2 Table of basic characteristics of studies on internet-based self-help interventions for individuals with Irritable Bowel Syndrome.

10.2196/87216 Multimedia Appendix 3 Subgroup analysis of all results, meta-regression bubble plots, sensitivity analysis plots, and funnel plots.

10.2196/87216 Checklist 1 PRISMA 2020 checklist.

## Abbreviations

CBM: China Biology Medicine Disc
CBT: cognitive behavioral therapy
CNKI: China National Knowledge Infrastructure
GRADE: Grading of Recommendations Assessment, Development and Evaluation
IBS: irritable bowel syndrome
PI: prediction interval
PRISMA: Preferred Reporting Items for Systematic Reviews and Meta-Analyses
RCT: randomized controlled trial
SMD: standardized mean difference
