# Supplementary material for: Efficacy of Internet-Based Self-Help Interventions for Irritable Bowel Syndrome: Systematic Review and Meta-Analysis of Randomized Controlled Trials
Source: J Med Internet Res. 2026 May 21;28:e87216. doi: 10.2196/87216 (PMC13193667; doi:10.2196/87216)
Supplement: Multimedia Appendix 1 [file jmir-v28-e87216-s001.docx]

**Appendix 2** Search strategy

| Database | Search strategy | Results |
| --- | --- | --- |
| Pubmed (NCBI) | ("irritable bowel syndromes"[Title/Abstract] OR "syndrome irritable bowel"[Title/Abstract] OR "syndromes irritable bowel"[Title/Abstract] OR "irritable colon"[Title/Abstract] OR "colon irritable"[Title/Abstract] OR "colitis mucous"[Title/Abstract] OR "mucous colitis"[Title/Abstract] OR "IBS"[Title/Abstract] OR "irritable bowel*"[Title/Abstract] OR "irritable bowel syndrome"[MeSH Terms]) AND ("internet"[MeSH Terms] OR "therapy, computer assisted"[MeSH Terms] OR "mobile applications"[MeSH Terms] OR "telemedicine"[MeSH Terms] OR "distance counseling"[MeSH Terms] OR "internet based intervention"[MeSH Terms] OR ("internet"[Title/Abstract] OR "mobile applications"[Title/Abstract] OR "telemedicine"[Title/Abstract] OR "distance counseling"[Title/Abstract] OR "ehealth"[Title/Abstract] OR "online*"[Title/Abstract] OR "web"[Title/Abstract] OR "tablet*"[Title/Abstract] OR "compute*"[Title/Abstract] OR "smartphone*"[Title/Abstract] OR "digital*"[Title/Abstract] OR "app"[Title/Abstract] OR "virtual"[Title/Abstract] OR "mhealth"[Title/Abstract] OR "web-based"[Title/Abstract] OR "internet-based"[Title/Abstract] OR "computer-based"[Title/Abstract] OR "mobile*"[Title/Abstract] OR "platform*"[Title/Abstract])) AND ("randomized controlled trial"[Publication Type] OR ("clinical trial"[Publication Type] OR "clinical trials as topic"[MeSH Terms] OR "clinical trial"[All Fields]) OR ("randomized controlled trial"[Publication Type] OR "randomized controlled trials as topic"[MeSH Terms] OR "randomized controlled trial"[All Fields] OR "randomised controlled trial"[All Fields]) OR ("controlled clinical trial"[Publication Type] OR "controlled clinical trials as topic"[MeSH Terms] OR "controlled clinical trial"[All Fields]) OR ("random allocation"[MeSH Terms] OR ("random"[All Fields] AND "allocation"[All Fields]) OR "random allocation"[All Fields]) OR "RCT"[All Fields]) | 314 |
| Embase | ('irritable bowel syndromes':ti,ab,kw OR 'syndrome irritable bowel':ti,ab,kw OR 'syndromes irritable bowel':ti,ab,kw OR 'irritable colon':ti,ab,kw OR 'colon irritable':ti,ab,kw OR 'colitis mucous':ti,ab,kw OR 'mucous colitis':ti,ab,kw OR 'ibs':ti,ab,kw OR 'irritable bowel*':ti,ab,kw OR 'irritable bowel syndrome'/exp) AND ('internet'/exp OR 'therapy, computer assisted'/exp OR 'mobile applications'/exp OR 'telemedicine'/exp OR 'distance counseling'/exp OR 'internet based intervention'/exp OR 'internet':ti,ab,kw OR 'mobile applications':ti,ab,kw OR 'telemedicine':ti,ab,kw OR 'distance counseling':ti,ab,kw OR 'ehealth':ti,ab,kw OR 'online*':ti,ab,kw OR 'web':ti,ab,kw OR 'tablet*':ti,ab,kw OR 'compute*':ti,ab,kw OR 'smartphone*':ti,ab,kw OR 'digital*':ti,ab,kw OR 'app':ti,ab,kw OR 'virtual':ti,ab,kw OR 'mhealth':ti,ab,kw OR 'web-based':ti,ab,kw OR 'internet-based':ti,ab,kw OR 'computer-based':ti,ab,kw OR 'mobile*':ti,ab,kw OR 'platform*':ti,ab,kw) AND ('randomized controlled trial'/exp OR 'clinical trial'/exp OR 'controlled clinical trial'/exp OR 'random allocation'/exp OR 'rct') | 840 |
| Web of Science | TS=(irritable bowel syndrome* OR syndrome, irritable bowel OR syndromes, irritable bowel OR irritable colon OR colon, irritable OR colitis, mucous OR mucous colitis OR IBS OR irritable bowel*) AND TS=(internet OR therapy, computer assisted OR mobile applications OR telemedicine OR distance counseling OR internet based intervention OR internet OR mobile applications OR telemedicine OR distance counseling OR ehealth OR online* OR web OR tablet* OR compute* OR smartphone* OR digital* OR app OR virtual OR mhealth OR web-based OR internet-based OR computer-based OR mobile* OR platform*) AND TS=(Randomized controlled trial OR clinical trial OR controlled clinical trial OR RCT) | 450 |
| CINAHL | XB (irritable bowel syndrome* OR syndrome, irritable bowel OR syndromes, irritable bowel OR irritable colon OR colon, irritable OR colitis, mucous OR mucous colitis OR IBS OR irritable bowel*) AND TX (internet OR therapy, computer assisted OR mobile applications OR telemedicine OR distance counseling OR internet based intervention OR internet OR mobile applications OR telemedicine OR distance counseling OR ehealth OR online* OR web OR tablet* OR compute* OR smartphone* OR digital* OR app OR virtual OR mhealth OR web-based OR internet-based OR computer-based OR mobile* OR platform*) AND TX (Randomized controlled trial OR clinical trial OR controlled clinical trial OR RCT) | 230 |
| Cochrane Library | #1 MeSH descriptor: [Irritable Bowel Syndrome] explode all trees  #2 (irritable bowel syndrome* OR syndrome, irritable bowel OR syndromes, irritable bowel OR irritable colon OR colon, irritable OR colitis, mucous OR mucous colitis OR IBS OR irritable bowel*):ti,ab,kw  #3 #1 OR #2  #4 MeSH descriptor: [Internet] explode all trees  #5 MeSH descriptor: [Therapy, Computer-Assisted] explode all trees  #6 MeSH descriptor: [Mobile Applications] explode all trees  #7 MeSH descriptor: [Telemedicine] explode all trees  #8 MeSH descriptor: [Distance Counseling] explode all trees  #9 MeSH descriptor: [Internet-Based Intervention] explode all trees  #10 (internet OR mobile applications OR telemedicine OR distance counseling OR ehealth OR online* OR web OR tablet* OR compute* OR smartphone* OR digital* OR app OR virtual OR mhealth OR web-based OR internet-based OR computer-based OR mobile* OR platform*):ti,ab,kw  #11 #4 OR #5 OR #6 OR #7 OR #8 OR #9 OR #10  #12 MeSH descriptor: [Randomized Controlled Trial] explode all trees  #13 (Randomized controlled trial OR clinical trial OR controlled clinical trial OR RCT):ti,ab,kw  #14 #12 OR #13  #15 #3 AND #11 AND #14 | 464 |
| PsycINFO (Ovid) | ((irritable bowel syndrome* or syndrome, irritable bowel or syndromes, irritable bowel or irritable colon or colon, irritable or colitis, mucous or mucous colitis or IBS or irritable bowel*) and (internet or therapy, computer assisted or mobile applications or telemedicine or distance counseling or internet based intervention or mobile applications or telemedicine or distance counseling or ehealth or online* or web or tablet* or compute* or smartphone* or digital* or app or virtual or mhealth or web-based or internet-based or computer-based or mobile* or platform*) and (Randomized controlled trial or clinical trial or controlled clinical trial or RCT)).ab,hw,id,ti | 28 |
| CNKI | SU=( 肠易激综合征 + IBS + 黏液性肠炎 + 肠功能紊乱 + 过敏性肠炎 ) AND SU=( 移动健康 + 移动应用 + APP + 网络 + 互联网 + 电子干预 + 电话 + 短信 + 电子邮件 + 远程 + 电脑 + 数字技术 ) AND FT=( RCT + 随机对照试验 + 随机 + 临床试验 ) | 366 |
| VIP | M=(肠易激综合征 OR IBS OR 黏液性肠炎 OR 肠功能紊乱 OR 过敏性肠炎) AND R=(移动健康 OR 移动应用 OR APP OR 网络 OR 互联网 OR 电子干预 OR 电话 OR 短信 OR 电子邮件 OR 远程 OR 电脑 OR 数字技术) AND R=(RCT OR 随机对照试验 OR 随机 OR 临床试验) | 51 |
| CBM | ("肠易激综合征"[不加权:扩展] OR "肠易激综合征"[常用字段:智能] OR "IBS"[常用字段:智能] OR "黏液性肠炎"[常用字段:智能] OR "肠功能紊乱"[常用字段:智能] OR "过敏性肠炎"[常用字段:智能]) AND ("远程医学"[不加权:扩展] OR "移动健康"[常用字段:智能] OR "移动应用"[常用字段:智能] OR "APP"[常用字段:智能] OR "网络"[常用字段:智能] OR "互联网"[常用字段:智能] OR "电子干预"[常用字段:智能] OR "电话"[常用字段:智能] OR "短信"[常用字段:智能] OR "电子邮件"[常用字段:智能] OR "远程"[常用字段:智能] OR "电脑"[常用字段:智能] OR "数字技术"[常用字段:智能]) AND ("随机对照试验"[不加权:扩展] OR "随机对照试验(主题)"[不加权:扩展] OR "RCT"[常用字段:智能] OR "随机对照试验"[常用字段:智能] OR "随机"[常用字段:智能] OR "临床试验"[常用字段:智能]) | 50 |
| WANGFANG | ((题名或关键词=(肠易激综合征 OR IBS OR 黏液性肠炎 OR 肠功能紊乱 OR 过敏性肠炎) AND 题名或关键词=(移动健康 OR 移动应用 OR APP OR 网络 OR 互联网 OR 电子干预 OR 电话 OR 短信 OR 电子邮件 OR 远程 OR 电脑 OR 数字技术)) AND (RCT OR 随机对照试验 OR 随机 OR 临床试验) | 18 |

Note: The last search dates for the above databases were all March 15, 2026.
