# Supplementary material for: Efficacy of Internet-Based Self-Help Interventions for Irritable Bowel Syndrome: Systematic Review and Meta-Analysis of Randomized Controlled Trials
Source: J Med Internet Res. 2026 May 21;28:e87216. doi: 10.2196/87216 (PMC13193667; doi:10.2196/87216)
Supplement: Multimedia Appendix 2 [file jmir-v28-e87216-s002.docx]

| **Author(s)**  **(year)** | **Participant Demographics[Country, Age (M±SD), Female]; Sample size (I/C)** | **Diagnostic criteria** | **Study location** | **Internet self-help intervention** | | | **control group** | **Outcome**  **measurements** |
| --- | --- | --- | --- | --- | --- | --- | --- | --- |
|  |  |  |  | **Intervention content** | **contact method** | **duration** |  |  |
| Anderson et al (2022) [1] | Country: US  Age: 37±32.9  Female: 90.4%  Sample size: 240 (121/119) | Rome IV | Multicenter | 1.Based on the reading materials related to the intestinal-brain axis for psychological education, cognitive-behavioral therapy techniques, and relaxation training such as diaphragmatic breathing;  2.The scripted intestinal directional hypnotherapy recordings developed by experienced clinical intestinal-oriented hypnotherapists. | Guided type with the lowest intensity contact | 42 daily treatment sessions (once a day);  The intervention group has a single session of approximately 10-15 minutes, while the control group has a single session of approximately 5-10 minutes;  Follow-up was conducted 6 months after the intervention. | Relaxation training guidance | 1. IBS SSS  2. IBS-QOL  3. DASS-21 |
| Chao et al (2024) [2] | Country: China  Age: 37.23±5.69  Female: 71.4%  Sample size: 21(10/11) | Rome IV | Single center | 1. Online yoga delivered via the internet;  2. 2 capsules of probiotics per day;  3.Smartwatch monitors exercise intensity. | Guided type with the lowest intensity contact | Intervention for 6 weeks; the intervention group received 3 times per week, while the probiotics were taken once daily. | Only 2 capsules of the same type of probiotic per day. | 1. IBS-QOL |
| D'Silva et al (2022) [3] | Country: Canada  Age: 45.4±13.9  Female: 92.4%  Sample size: 79 (38/41) | Rome IV | Single center | 1.Use online platforms for yoga intervention training  2.Family video practice  3.Keep a weekly practice log | Guided type with the lowest intensity contact | 8 weeks (once a week, 60 minutes each time) | Placebo (Video exercise) | 1. IBS-SSS  2. IBS-QOL  3. GAD-7  4. PHQ-9 |
| Everitt et al (2013) [4] | Country: UK  Age: 44.0±10.5  Female: 78.9%  Sample size: 90 (45/45) | Rome III | Multicenter | 1.Web-based cognitive behavioral therapy self-management project  2.Provide minimum email support as needed | Guided type with the lowest intensity contact | 6 weeks  Follow-up for 12 weeks | Usual care | 1. IBS-SSS  2. IBS-QOL  4. HADS-A  5. HADS-D |
| Everitt et al (2019) [5] | Country: UK  Age: 42.9±13.6  Female: 76.3%  Sample size: 372 (185/187) | ROME III | Multicenter | 1.Low-intensity online cognitive behavioral therapy  2.Combined with telephone therapy  3.Intensive Courses | Guided type with the lowest intensity contact | 8 months and 9 weeks (8 online courses, 3 30-minute phone therapy sessions, and 2 30-minute intensive courses) | Usual care | 1. IBS-SSS |
| Hunt et al (2009) [6] | Country: US  Age: 38.5±10.9  Female: 81.5%  Sample size: 54 (28/26) | Rome II | Single center | 1.Cognitive behavioral therapy in the network module  2.Email feedback  3.Fill in the symptom list every week | Guided type with the lowest intensity contact | 5 weeks (including five modules, one per week)  Follow-up for 3 months | Waiting list | 1. GSRS-IBS  2. IBS–QOL |
| Hunt et al (2021) [7] | Country: US  Age: 32±10.2  Female: 75.2%  Sample size: 121 (62/59) | Rome III | Single center | 1.Conduct digital cognitive behavioral therapy through mobile applications  2.Chatbots guide the completion of tasks | Unguided | 8 weeks (10 modules in total, 1-2 modules per week)  Follow-up for 3 months | Waiting list | 1. GSRS-IBS  2. IBS-QOL  3. VSI  4. PHQ-9 |
| Hunt et al (2025) [8] | Country: US  Age: 36.56±13.24  Female: 72.3%  Sample size: 267 (136/131) | Rome III | Single center | 1.6 Digital online delivery, core modules, including psychological education on the gut-brain axis, abdominal breathing training, motivational interviewing, cognitive therapy, exposure therapy, reintroduction of feared foods, and prevention of relapse;  2. Additional tools include intestinal-targeted hypnosis scripts, mindfulness exercises, yoga videos;  3. Support the weekly push notification function. | Guided type with the lowest intensity contact | 8 weeks  Follow-up for 3 months and 6 months | Active Control App | 1. GSRS-IBS  2. IBS-QOL  3. VSI  4. BDI |
| Lee et al (2019) [9] | Country: China  Age: 18.53  Female: 100%  Sample size: 118 (48/70) | Rome III | Single center | 1.Use standardized self-service materials provided by the Internet for cognitive behavioral therapy  2.Homework  3.Hold individual meetings | Guided type with the lowest intensity contact | 6 weeks (13 online courses, 3 modules  Follow-up for 3 months | Written expression | 1. IBS-SSS  2. STAI-S  3. CES-D |
| Lindfors et al (2020) [10] | Country: Sweden  Age: 37.0±15.0  Female: 80.1%  Sample size: 141 (71/70) | Rome IV | Single center | 1.Provide standardized electronic materials through online platforms  2.Provide limited professional exposure  3.Peer Interaction | Guided type with the lowest intensity contact | 3 weeks (one module per week)  Follow up for 6 months | Face-to-face education | 1. IBS-SSS  2. IBS-QOL  3. VSI  4. HADS-A  5. HADS-D |
| Ljótsson et al (2010) [11] | Country: Sweden  Age: 34.6±9.4  Female: 84.7%  Sample size: 85 (42/43) | Rome III | Multicenter | 1.Internet-based cognitive behavioral therapy based on exposure and mindfulness  2.Online feedback  3.Online discussion forum  4.Keep a symptom diary | Guided type with the lowest intensity contact | 10 weeks (a total of 5 steps, one per week, and the fifth step lasts for 5 weeks)  Follow-up for 3 months | Waiting list | 1. GSRS-IBS  2. IBS-QOL  3. VSI  4. MADRS-S |
| Ljotsson et al (2011 a) [12] | Country: Sweden  Age: 34.9±11.3  Female: 74.0%  Sample size: 61 (30/31) | Rome III | Single center | 1.Internet-based cognitive behavioral therapy  2.Online treatment manual combined with videos  3.Online Discussion Forum | Guided type with the lowest intensity contact | 10 weeks (a total of 5 steps, one per week, and the fifth step lasts for 5 weeks)  Follow-up for 12 months | Waiting list | 1. GSRS-IBS  2. IBS-QOL  3. VSI |
| Ljotsson et al (2011 b) [13] | Country: Sweden  Age: 37.85±11.11  Female: 79.0%  Sample size: 195 (98/97) | Rome III | Multicenter | 1.Internet-based cognitive behavioral therapy focuses on mindfulness exercises, exposure therapy, symptom control behaviors reduction, etc., for gastrointestinal symptoms.  2.Online therapist communication. | Guided type with the lowest intensity contact | 10 weeks  Follow-up for 6 months | internet-delivered stress management | 1. GSRS-IBS  2. IBS-QOL  3. VSI  4. HADS-A  5. HADS-D |
| Ljótsson et al (2014) [14] | Country: Sweden  Age: 42.44±14.5  Female: 76.1%  Sample size: 309 (153/156) | Rome III | Multicenter | 1.Internet-based cognitive behavioral therapy;  2.Based on mindfulness, acceptance, and value-oriented behavioral change;  3.Increase systematic exposure training for IBS; 4.Online therapist communication | Structured guidance | 10 weeks  Follow-up for 6 months | Internet-based cognitive behavioral therapy without exposure | 1. GSRS-IBS  2. IBS-QOL  3. VSI  4. HADS-A  5. HADS-D |
| Owusu et al (2020) [15] | Country: US  Age: 39.2±11.9  Female: 77.8%  Sample size: 36 (25/11) | Rome IV | Multicenter | 1.Cognitive behavioral therapy in the network module  2.The application guides the recording of symptoms and the completion of assignments | Unguided | 12 weeks (including 8 modules, with automatic email reminders sent every 2 weeks) | Usual care | 1. IBS-SSS  2. PHQ-9  3. GAD-7 |
| Tayama et al (2024) [16] | Country: Japan  Age: 21.1±2.8  Female: 100%  Sample size: 40 (21/19) | Rome IV | Multicenter | 1.Use the e-health program for self-management  2.Including video and electronic text formats | Unguided | 8 weeks (once a week, 60 minutes each time) | Usual care | 1. IBS-SSS  2. IBS-QoL |
| Zargar et al (2023) [17] | Country: Iran  Age: 33.1±4.7  Female: 56.7%  Sample size: 60 (30/30) | Rome III | Single center | 1.Internet-delivered  progressive muscle relaxation  2.Followed through  WhatsApp  3.Psychoeducation | Guided type with the lowest intensity contact | 4 weeks  Follow-up for 2 months | psychoeducation | 1. GSRS-IBS  2. VSI |

Note: IBS-SSS = IBS Symptom Severity Scale, IBS-QOL = IBS Quality of life, DASS-21= Depression Anxiety and Stress Scale-21, GAD-7 = Generalized Anxiety Disorder-7, PHQ-9 = Patient Health Questionnaire-9, GSRS-IBS = Gastrointestinal Symptom Rating Scale - IBS, HADS-A = Hospital Anxiety and Depression Scale for anxiety, HADS-D = Hospital Anxiety and Depression Scale for depression, VSI = Visceral Sensitivity Index, MADRS-S = The Montgomery Asberg Depression Rating Scale-Self, BSSS = Bowel Symptom Severity Scale, STAI-S = State-Trait Anxiety Inventory, CES-D = Center for Epidemiological Studies Depression Scal.

**References:**

1. Anderson EJ, Peters SL, Gibson PR, Halmos EP. Comparison of Digitally Delivered Gut-Directed Hypnotherapy Program With an Active Control for Irritable Bowel Syndrome. Am J Gastroenterol. 2025 Feb 1;120(2):440-8. PMID: 38940439. doi: 10.14309/ajg.0000000000002921.

2. Chao WC, Huang JC, Young SL, Wu CL, Shih JC, Liao LD, et al. Interplay of yoga, physical activity, and probiotics in irritable bowel syndrome management: A double-blind randomized study. Complementary Therapies in Clinical Practice. 2024;57(000):7. PMID: 39126817. doi: 10.1016/j.ctcp.2024.101892.

3. D'Silva A, Marshall DA, Vallance JK, Nasser Y, Rajagopalan V, Szostakiwskyj JH, et al. Meditation and Yoga for Irritable Bowel Syndrome: A Randomized Clinical Trial. Am J Gastroenterol. 2023 Feb 1;118(2):329-37. PMID: 36422517. doi: 10.14309/ajg.0000000000002052.

4. Everitt H, Moss-Morris R, Sibelli A, Tapp L, Coleman N, Yardley L, et al. Management of irritable bowel syndrome in primary care: the results of an exploratory randomised controlled trial of mebeverine, methylcellulose, placebo and a self-management website. BMC Gastroenterol. 2013 Apr 21;13. PMID: WOS:000318682000001. doi: 10.1186/1471-230x-13-68.

5. Everitt HA, Landau S, O'Reilly G, Sibelli A, Hughes S, Windgassen S, et al. Assessing telephone-delivered cognitive-behavioural therapy (CBT) and web-delivered CBT versus treatment as usual in irritable bowel syndrome (ACTIB): a multicentre randomised trial. Gut. 2019 Sep;68(9):1613-23. PMID: 30971419. doi: 10.1136/gutjnl-2018-317805.

6. Hunt MG, Moshier S, Milonova M. Brief cognitive-behavioral internet therapy for irritable bowel syndrome. Behav Res Ther. 2009 Sep;47(9):797-802. PMID: 19570525. doi: 10.1016/j.brat.2009.05.002.

7. Hunt M, Miguez S, Dukas B, Onwude O, White S. Efficacy of Zemedy, a Mobile Digital Therapeutic for the Self-management of Irritable Bowel Syndrome: crossover Randomized Controlled Trial. JMIR mHealth and uHealth. 2021;9(5):e26152. PMID: CN-02273123. doi: 10.2196/26152.

8. Hunt M, Dalvie A, Ipek S, Glinski S, Macks R. Efficacy of a CBT Self-Help App (Zemedy) Versus an Education, Relaxation, and Mindfulness App for IBS: results from Post-Treatment, 3-Month, and 6-Month Follow-Up. Journal of clinical gastroenterology. 2025. PMID: CN-02841105. doi: 10.1097/MCG.0000000000002164.

9. Lee TY, Hsieh TC, Sung HC, Chen WL. Internet-Delivered Cognitive Behavior Therapy for Young Taiwanese Female Nursing Students with Irritable Bowel Syndrome-A Cluster Randomized Controlled Trial. Int J Environ Res Public Health. 2019 Feb 27;16(5). PMID: 30818837. doi: 10.3390/ijerph16050708.

10. Lindfors P, Axelsson E, Engstrand K, Störsrud S, Jerlstad P, Törnblom H, et al. Online Education Is Non-Inferior to Group Education for Irritable Bowel Syndrome: a Randomized Trial and Patient Preference Trial. Clin Gastroenterol Hepatol. 2020;19(4):743‐51.e1. PMID: CN-02099449. doi: 10.1016/j.cgh.2020.04.005.

11. Ljótsson B, Falk L, Vesterlund AW, Hedman E, Lindfors P, Rück C, et al. Internet-delivered exposure and mindfulness based therapy for irritable bowel syndrome - A randomized controlled trial. Behav Res Ther. 2010;48(6):531-9. PMID: 20362976. doi: 10.1016/j.brat.2010.03.003.

12. Ljotsson B, Andersson G, Andersson E, Hedman E, Lindfors P, Andreewitch S, et al. Acceptability, effectiveness, and cost-effectiveness of internet-based exposure treatment for irritable bowel syndrome in a clinical sample: a randomized controlled trial. BMC Gastroenterol. 2011 Oct 12;11. PMID: WOS:000296617700001. doi: 10.1186/1471-230x-11-110.

13. Ljótsson B, Hedman E, Andersson E, Hesser H, Lindfors P, Hursti T, et al. Internet-delivered exposure-based treatment vs. stress management for irritable bowel syndrome: a randomized trial. The American Journal of Gastroenterology. 2011;106(8):1481-91. PMID: 21537360. doi: 10.1038/ajg.2011.139.

14. Ljótsson Bn, Hesser H, Andersson E, Lackner JM, El Alaoui S, Falk L, et al. Provoking symptoms to relieve symptoms: A randomized controlled dismantling study of exposure therapy in irritable bowel syndrome. BEHAVIOUR RESEARCH AND THERAPY. 2014. PMID: 24584055. doi: 10.1016/j.brat.2014.01.007.

15. Owusu JT, Sibelli A, Moss-Morris R, van Tilburg MAL, Levy RL, Oser M. A pilot feasibility study of an unguided, internet-delivered cognitive behavioral therapy program for irritable bowel syndrome. Neurogastroenterol Motil. 2021 Nov;33(11):e14108. PMID: 33745228. doi: 10.1111/nmo.14108.

16. Tayama J, Hamaguchi T, Koizumi K, Yamamura R, Okubo R, Kawahara J-i, et al. Efficacy of an eHealth self-management program in reducing irritable bowel syndrome symptom severity: a randomized controlled trial. Scientific Reports. 2024 Jan 3;14(1). PMID: WOS:001145989400090. doi: 10.1038/s41598-023-50293-z.

17. Zargar F, Fahim A, Nikgoftar N, Tarrahi MJ. Comparing the effect of internet-delivered short-term progressive muscle relaxation and psychoeducation on mindful ability, visceral hypersensitivity and symptoms of patients with irritable bowel syndrome. J Educ Health Promot. 2023;12:259. PMID: 37727438. doi: 10.4103/jehp.jehp_1734_22.
