# Supplementary material for: Efficacy of Internet-Based Self-Help Interventions for Irritable Bowel Syndrome: Systematic Review and Meta-Analysis of Randomized Controlled Trials
Source: J Med Internet Res. 2026 May 21;28:e87216. doi: 10.2196/87216 (PMC13193667; doi:10.2196/87216)
Supplement: Multimedia Appendix 3 [file jmir-v28-e87216-s003.docx]

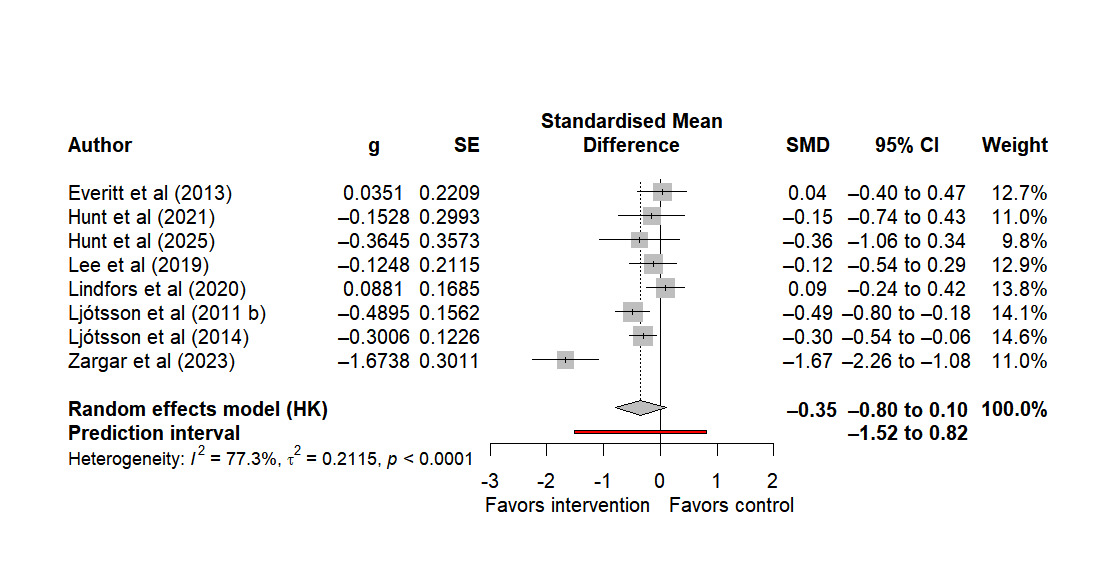


**Figure S1.** Forest plot of the severity of IBS symptoms results in follow-up assessments.


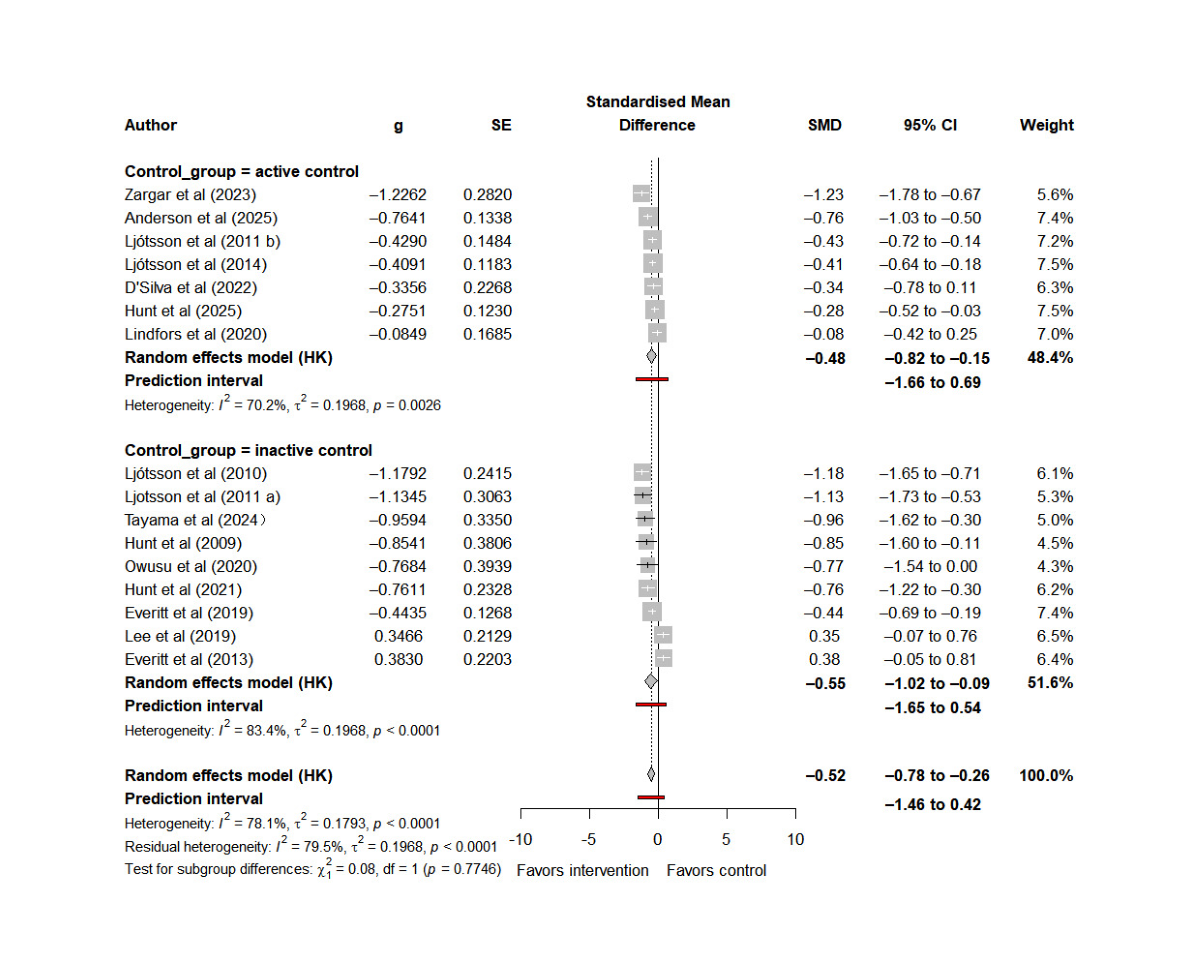


**Figure S2.** Forest plot of the severity of IBS symptoms results in the control group type subgroup analysis.


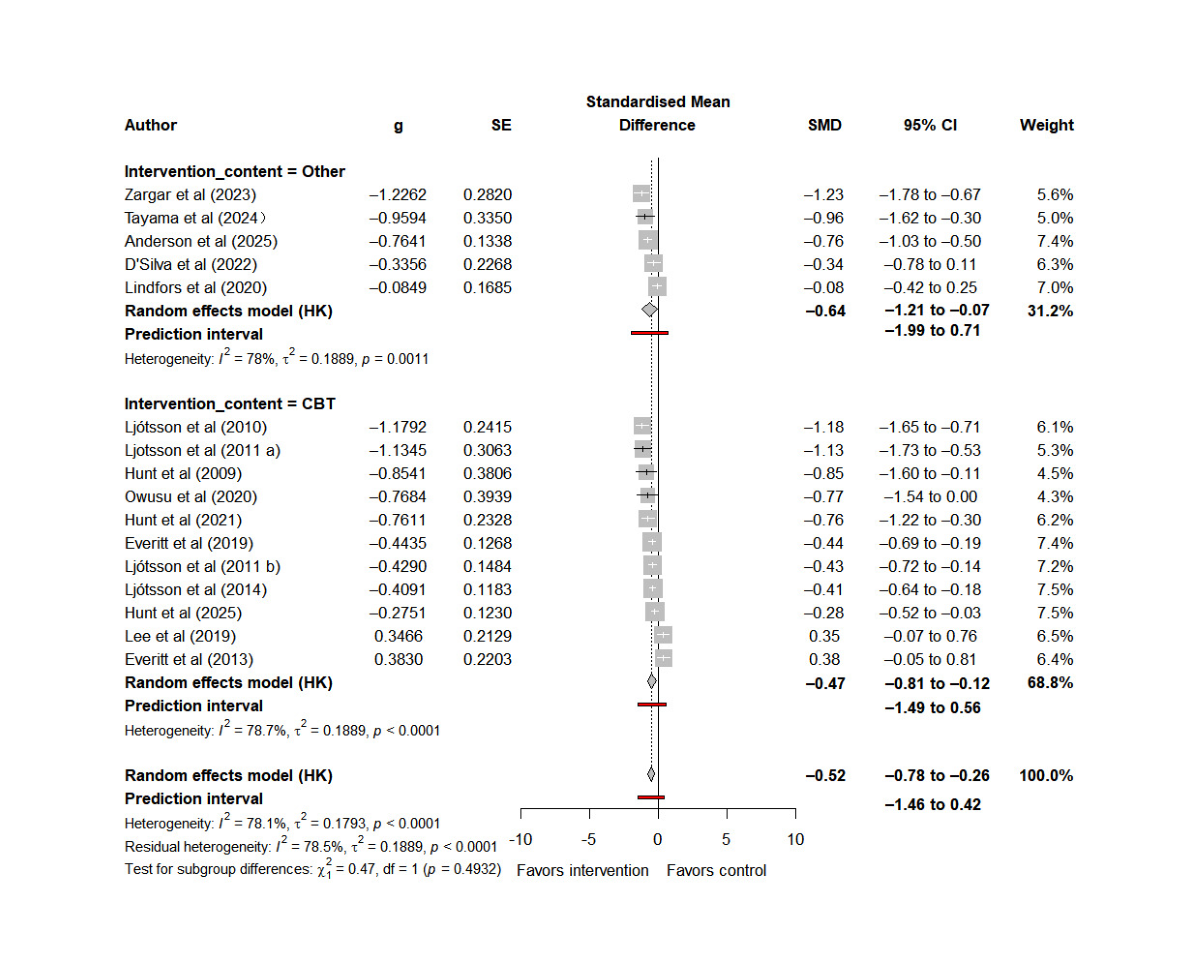


**Figure S3.** Forest plot of the severity of IBS symptoms results in the intervention content subgroup analysis.


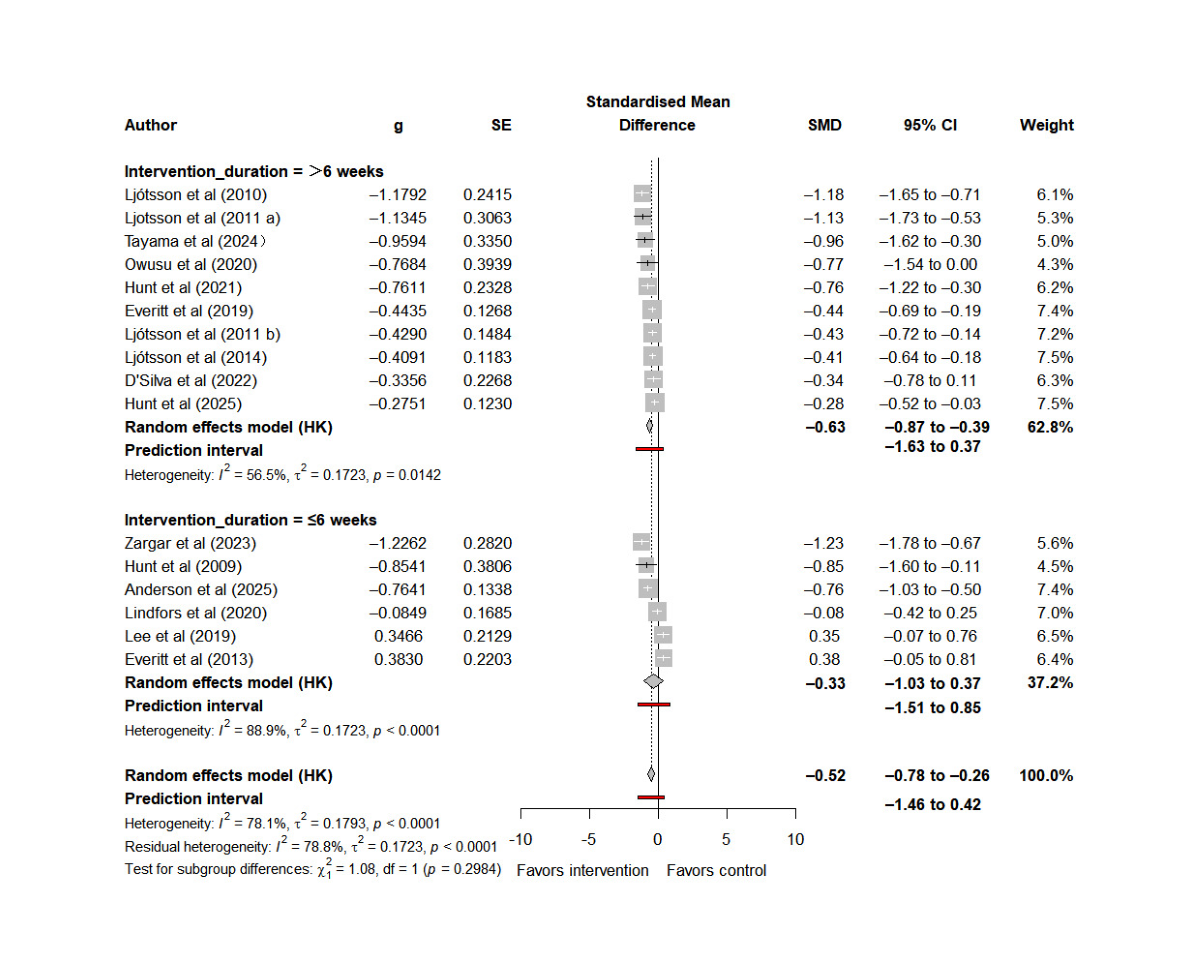


**Figure S4.** Forest plot of the severity of IBS symptoms results in the intervention duration subgroup analysis.


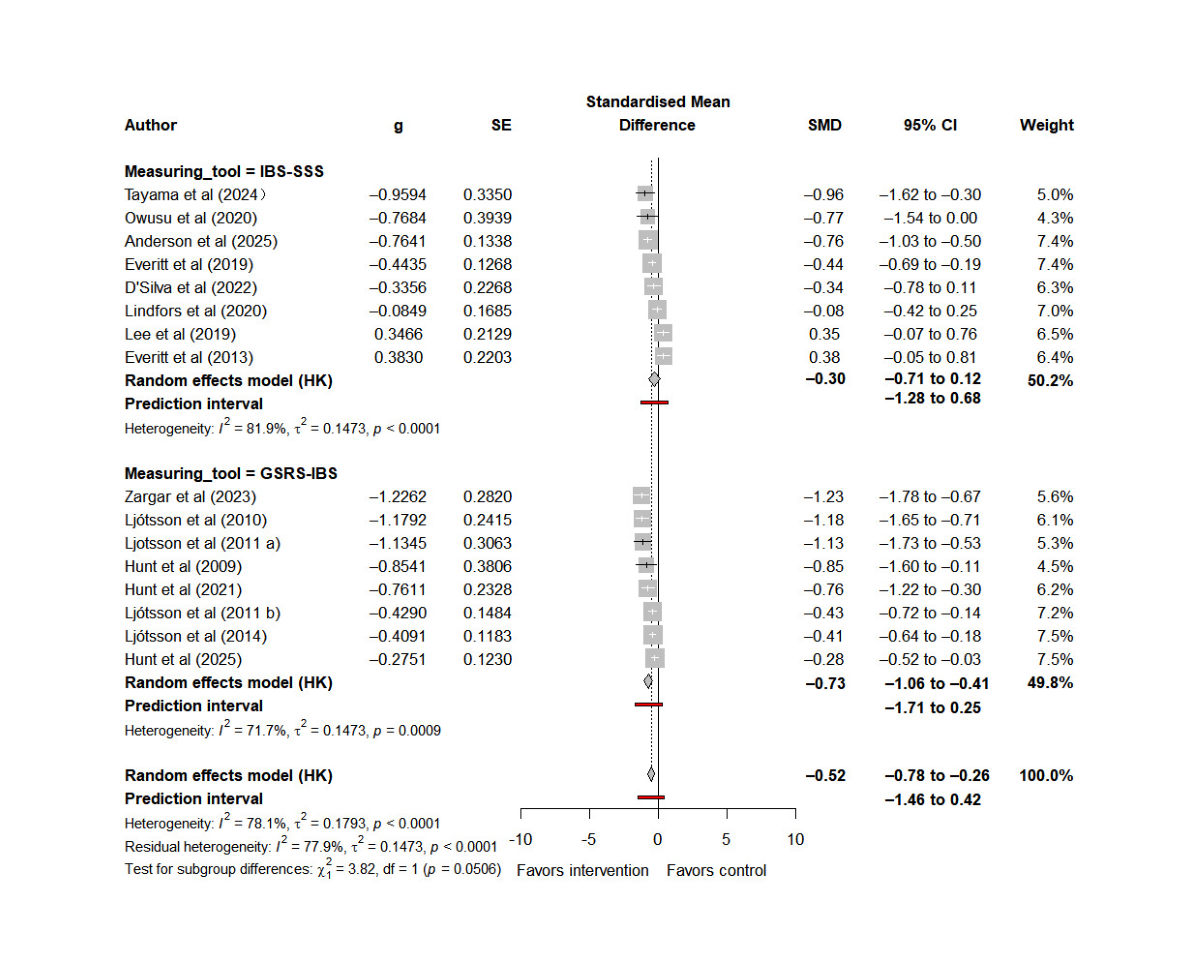


**Figure S5.** Forest plot of the severity of IBS symptoms results from the measuring tool subgroup analysis.


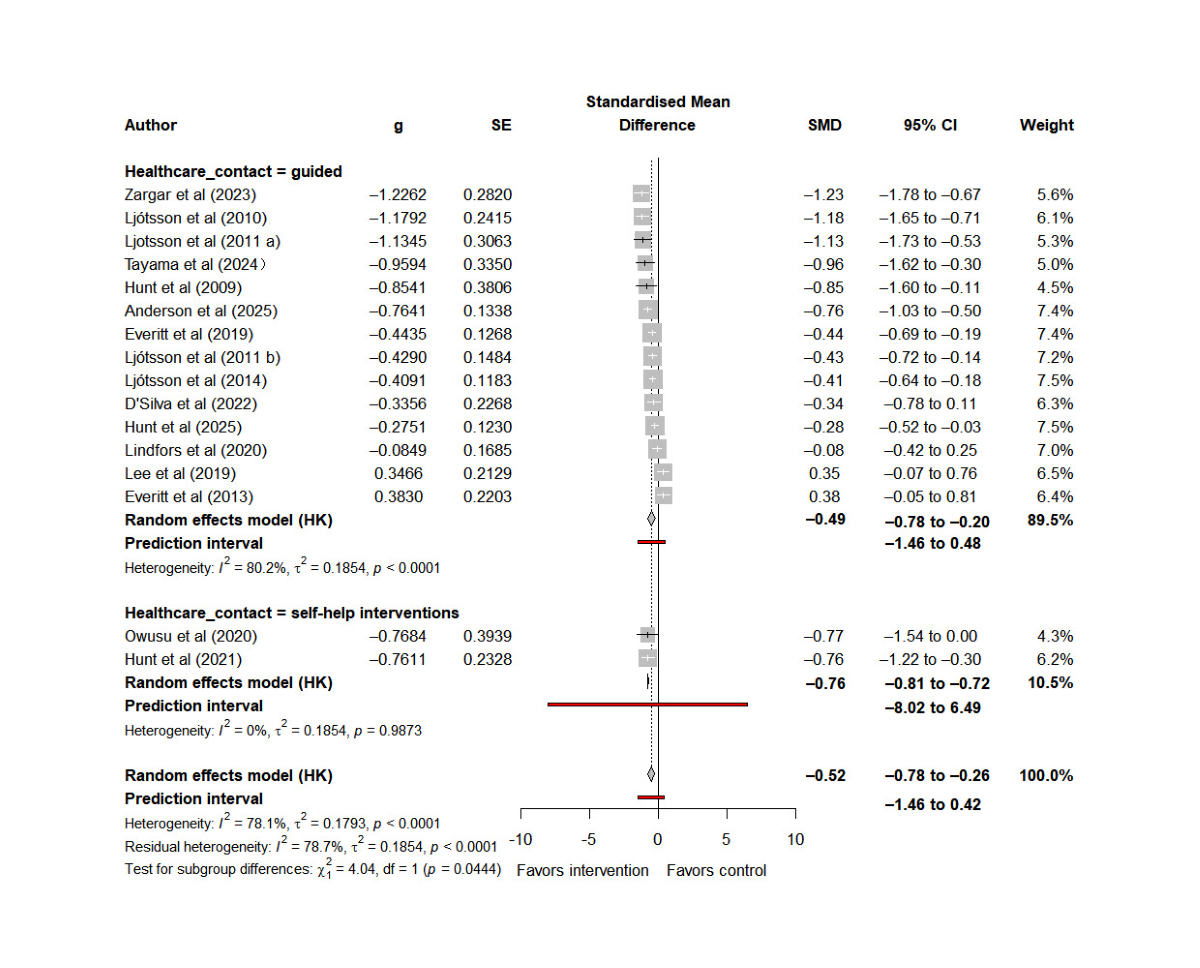


**Figure S6.** Forest plot of the severity of IBS symptoms results in the healthcare contact subgroup analysis.


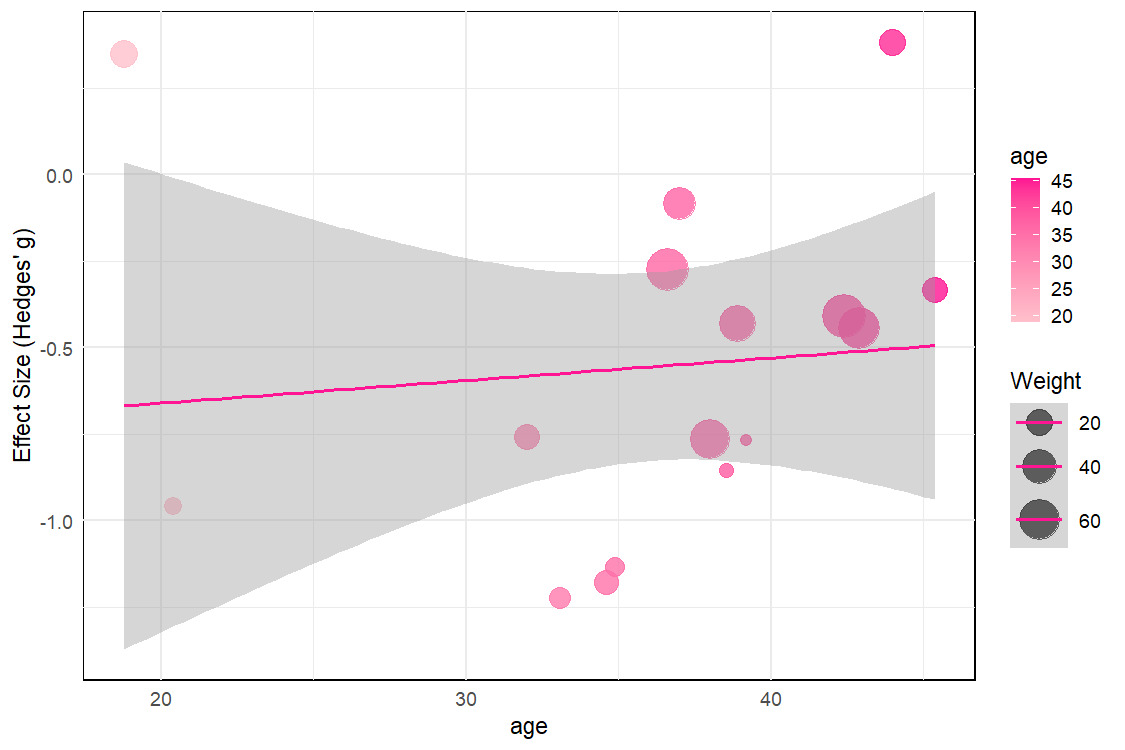


**Figure S7.** Bubble plot of meta-regression the severity of IBS symptoms results by age.


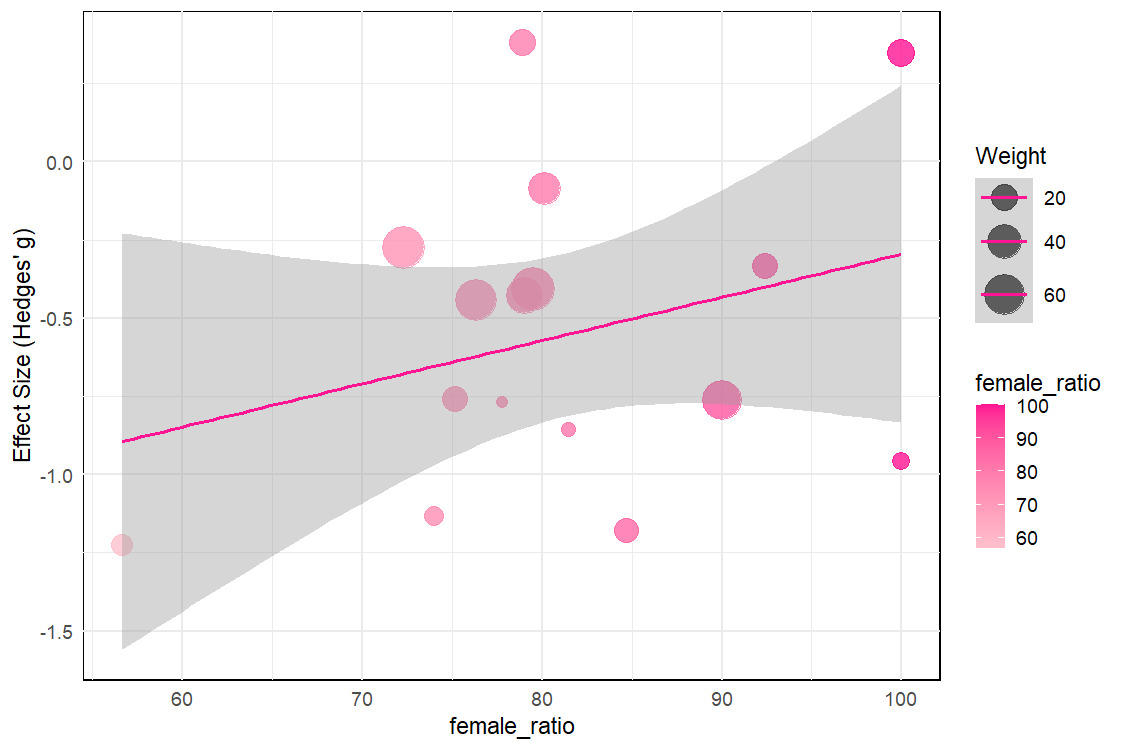


**Figure S8.** Bubble plot of meta-regression the severity of IBS symptoms results by female ratio.


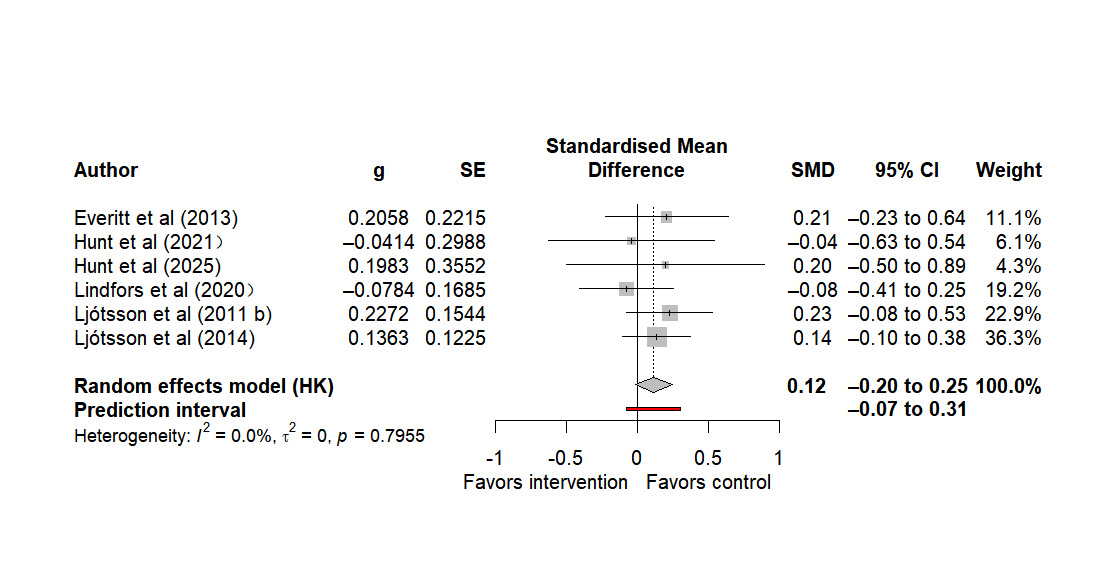


**Figure S9.** Forest plot of the quality of life results in follow-up assessments.


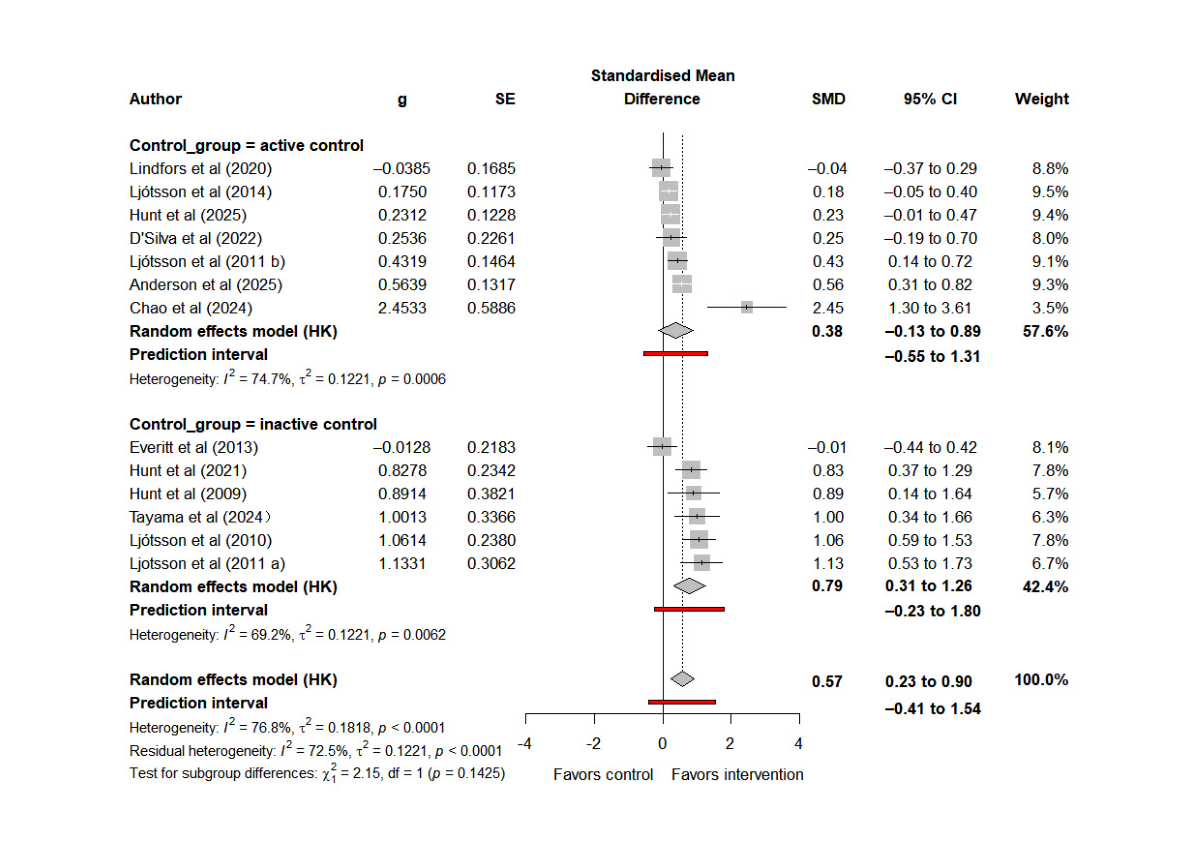


**Figure S10.** Forest plot of the quality of life results in the control group type subgroup analysis.


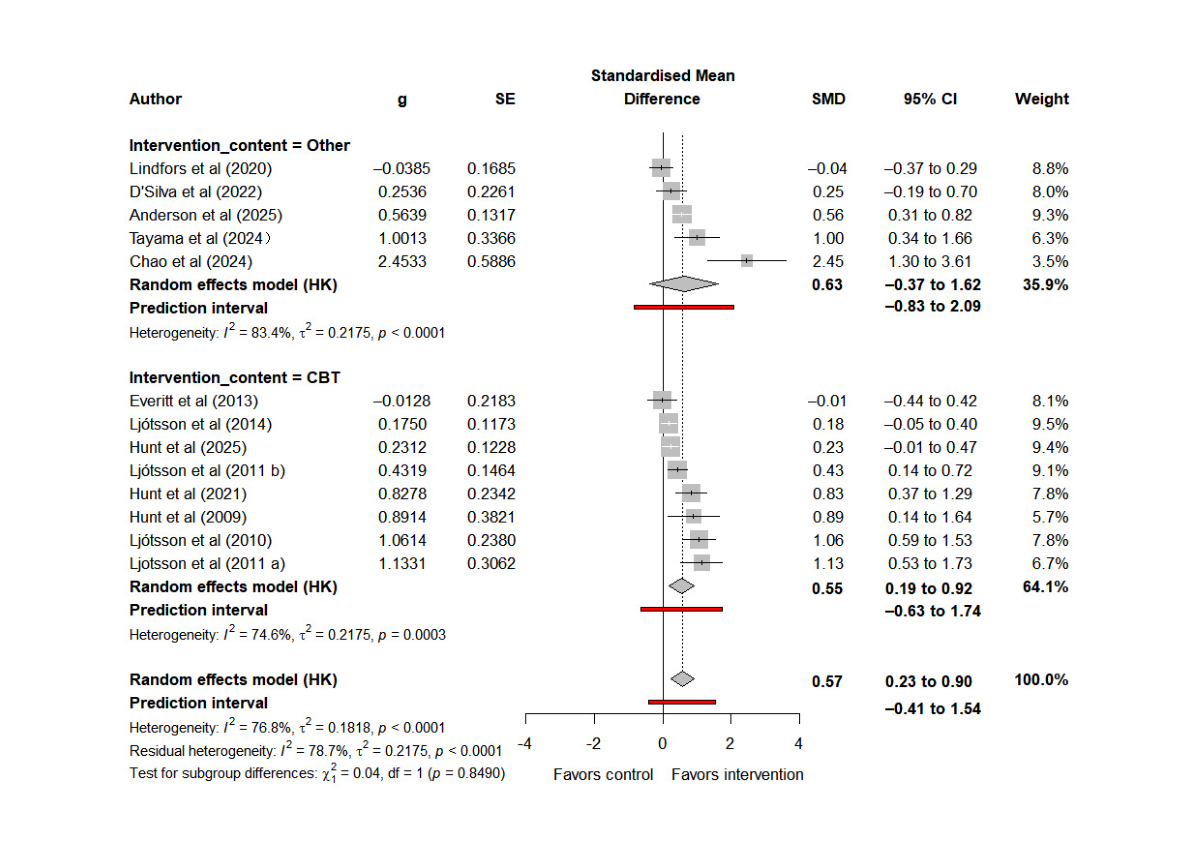


**Figure S11.** Forest plot of the quality of life results in the intervention content subgroup analysis.


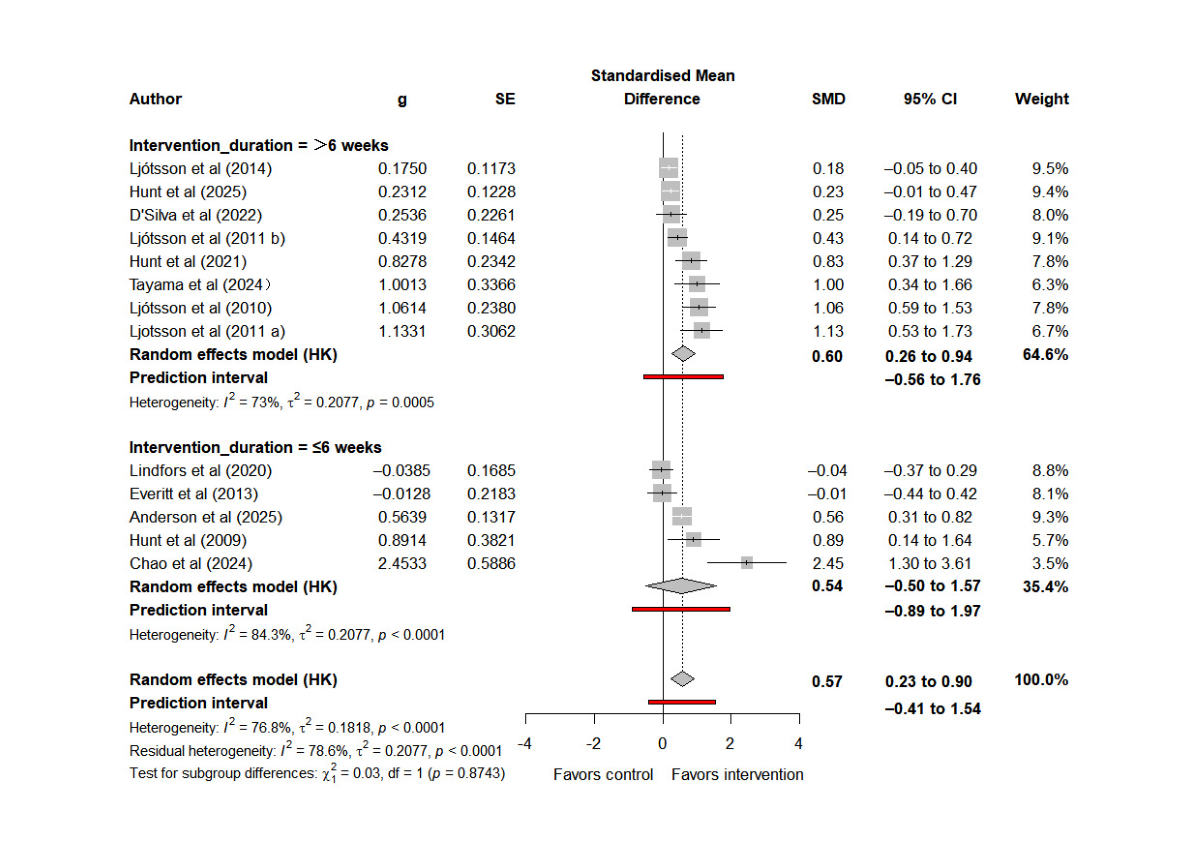


**Figure S12.** Forest plot of the quality of life results in the intervention duration subgroup analysis.


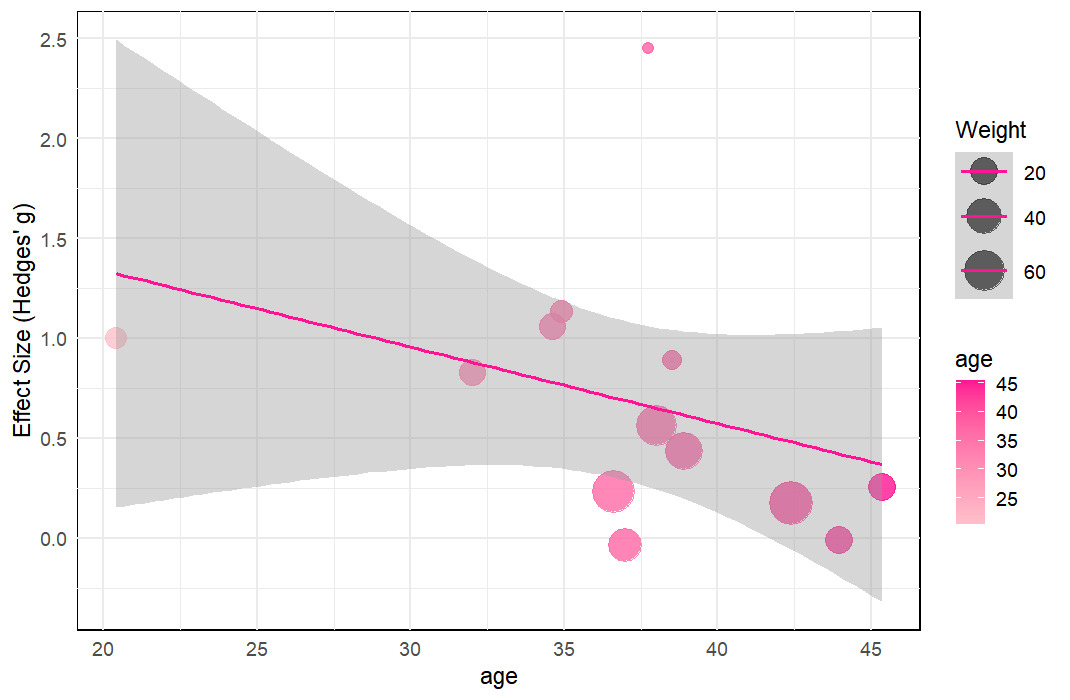


**Figure S13.** Bubble plot of meta-regression the quality of life results by age.


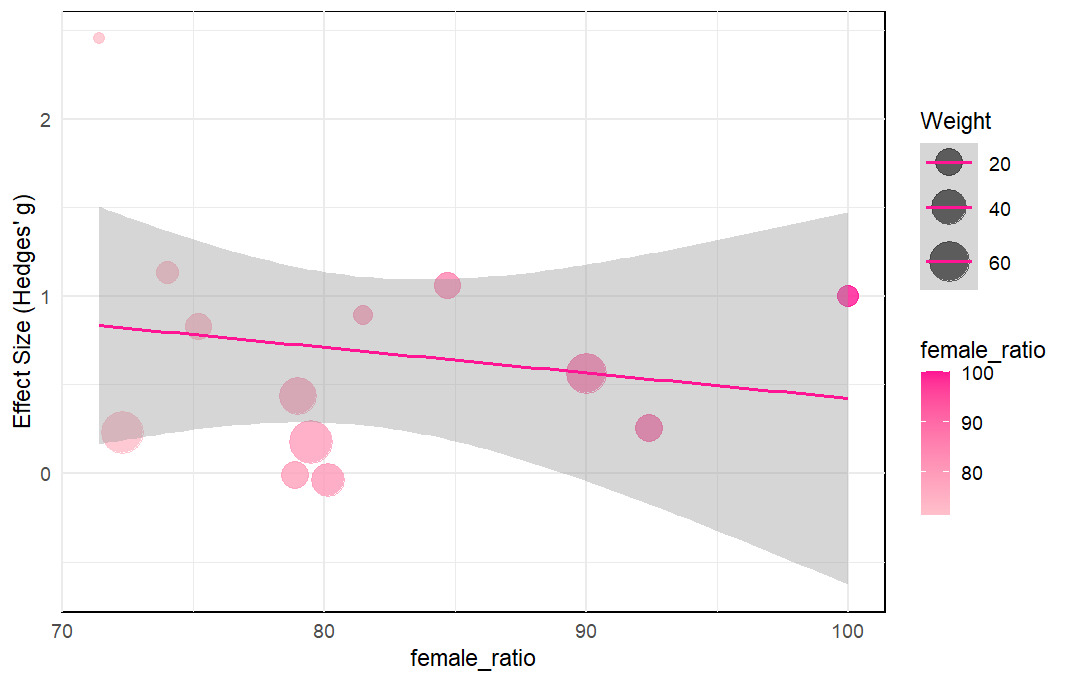


**Figure S14.** Bubble plot of meta-regression the quality of life results by female ratio.


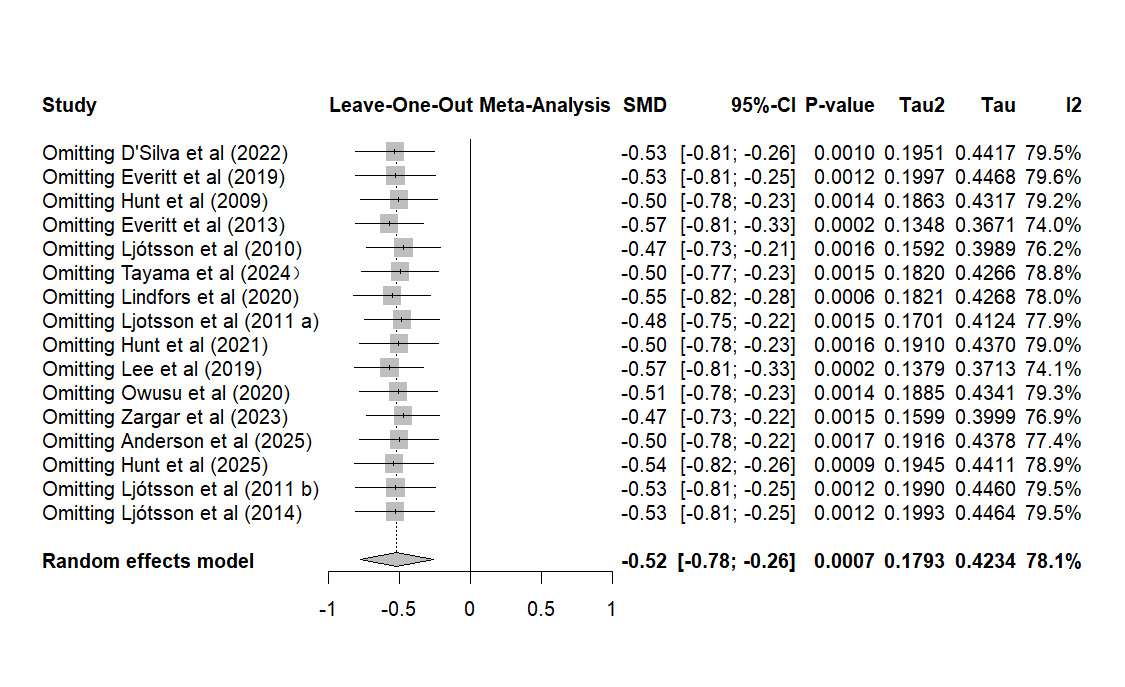


**Figure S15.** The results of the sensitivity analysis for severity of IBS symptoms.


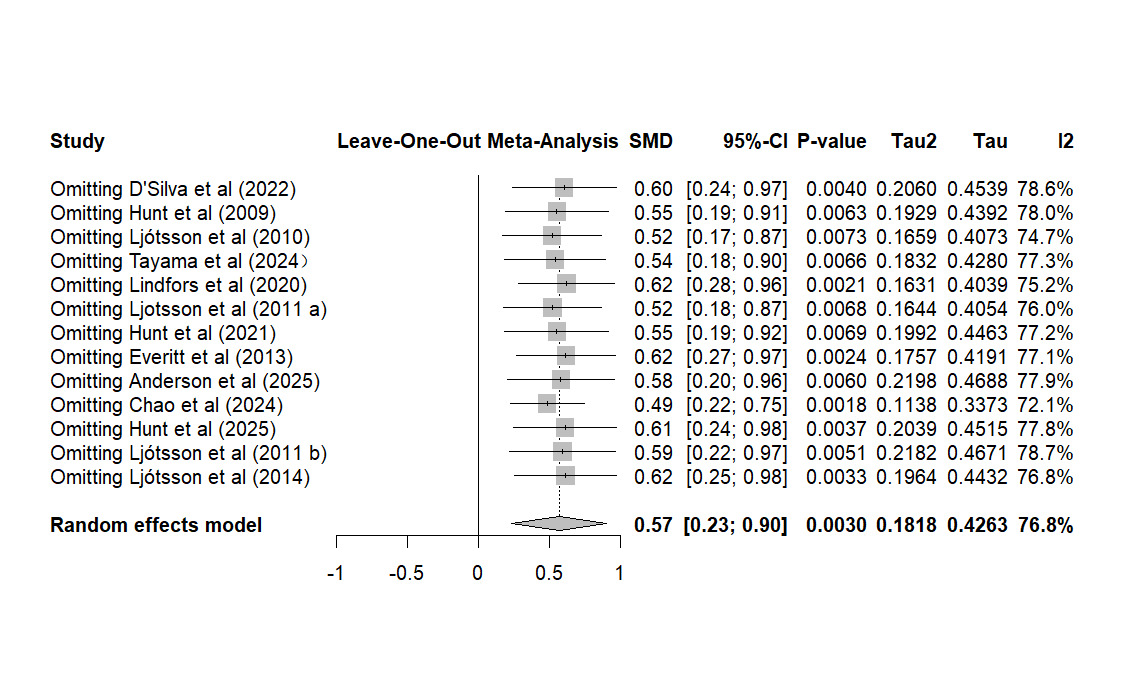


**Figure S16.** The results of the sensitivity analysis for quality of life.


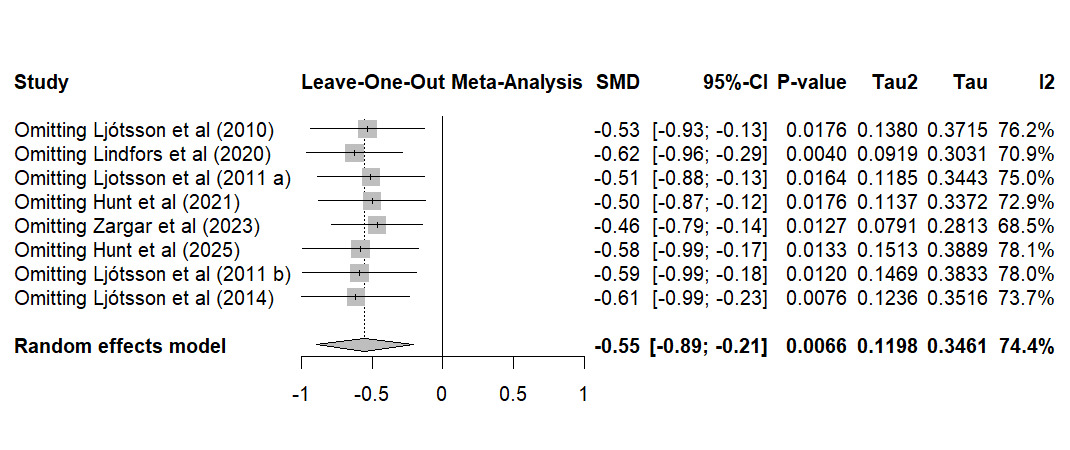


**Figure S17.** The results of the sensitivity analysis for visceral sensitivity.


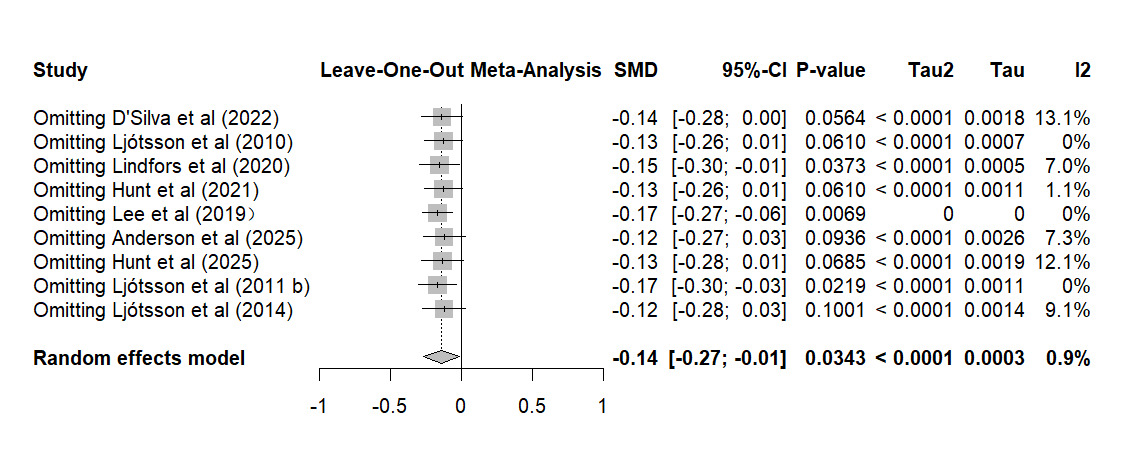


**Figure S18.**The results of the sensitivity analysis for depression.


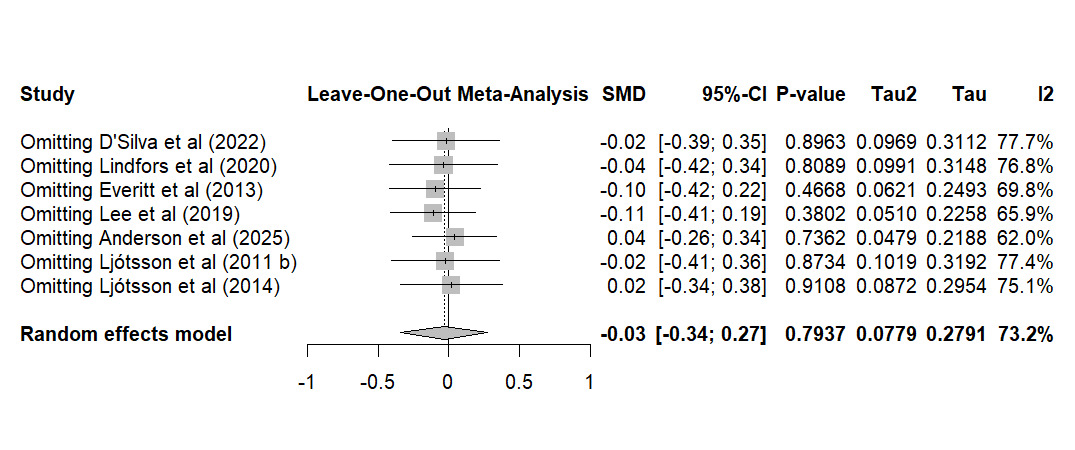


**Figure S19.** The results of the sensitivity analysis for anxiety.

**
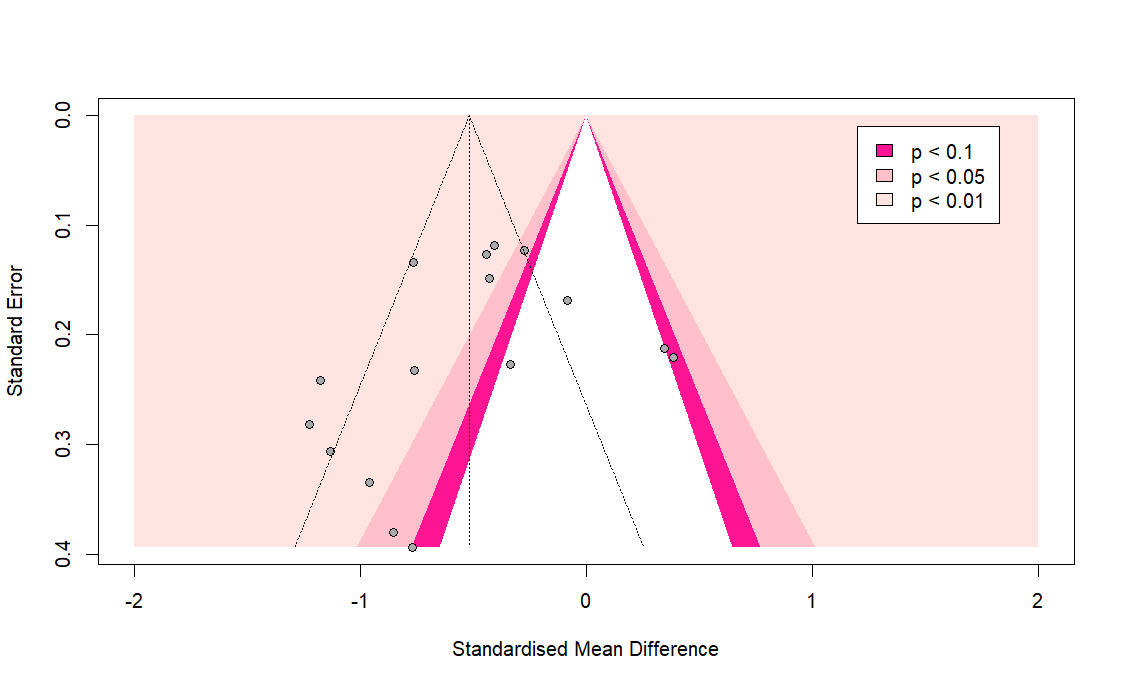
**

**Figure S20.** Funnel plot of effect sizes from small studies on IBS symptom severity in meta-analysis.

**
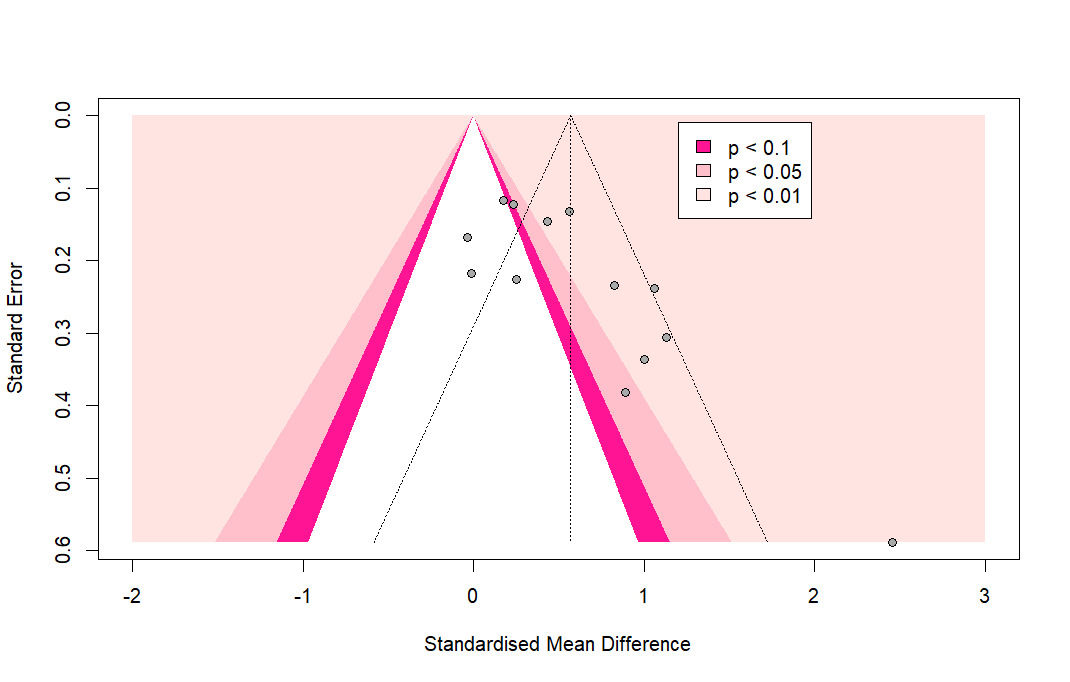
**

**Figure S21.** Funnel plot of small study effects on quality of life in meta-analysis.

**References:**

1. Anderson EJ, Peters SL, Gibson PR, Halmos EP. Comparison of Digitally Delivered Gut-Directed Hypnotherapy Program With an Active Control for Irritable Bowel Syndrome. Am J Gastroenterol. 2025 Feb 1;120(2):440-8. PMID: 38940439. doi: 10.14309/ajg.0000000000002921.

2. Chao WC, Huang JC, Young SL, Wu CL, Shih JC, Liao LD, et al. Interplay of yoga, physical activity, and probiotics in irritable bowel syndrome management: A double-blind randomized study. Complementary Therapies in Clinical Practice. 2024;57(000):7. PMID: 39126817. doi: 10.1016/j.ctcp.2024.101892.

3. D'Silva A, Marshall DA, Vallance JK, Nasser Y, Rajagopalan V, Szostakiwskyj JH, et al. Meditation and Yoga for Irritable Bowel Syndrome: A Randomized Clinical Trial. Am J Gastroenterol. 2023 Feb 1;118(2):329-37. PMID: 36422517. doi: 10.14309/ajg.0000000000002052.

4. Everitt H, Moss-Morris R, Sibelli A, Tapp L, Coleman N, Yardley L, et al. Management of irritable bowel syndrome in primary care: the results of an exploratory randomised controlled trial of mebeverine, methylcellulose, placebo and a self-management website. BMC Gastroenterol. 2013 Apr 21;13. PMID: WOS:000318682000001. doi: 10.1186/1471-230x-13-68.

5. Everitt HA, Landau S, O'Reilly G, Sibelli A, Hughes S, Windgassen S, et al. Assessing telephone-delivered cognitive-behavioural therapy (CBT) and web-delivered CBT versus treatment as usual in irritable bowel syndrome (ACTIB): a multicentre randomised trial. Gut. 2019 Sep;68(9):1613-23. PMID: 30971419. doi: 10.1136/gutjnl-2018-317805.

6. Hunt MG, Moshier S, Milonova M. Brief cognitive-behavioral internet therapy for irritable bowel syndrome. Behav Res Ther. 2009 Sep;47(9):797-802. PMID: 19570525. doi: 10.1016/j.brat.2009.05.002.

7. Hunt M, Miguez S, Dukas B, Onwude O, White S. Efficacy of Zemedy, a Mobile Digital Therapeutic for the Self-management of Irritable Bowel Syndrome: crossover Randomized Controlled Trial. JMIR mHealth and uHealth. 2021;9(5):e26152. PMID: CN-02273123. doi: 10.2196/26152.

8. Hunt M, Dalvie A, Ipek S, Glinski S, Macks R. Efficacy of a CBT Self-Help App (Zemedy) Versus an Education, Relaxation, and Mindfulness App for IBS: results from Post-Treatment, 3-Month, and 6-Month Follow-Up. Journal of clinical gastroenterology. 2025. PMID: CN-02841105. doi: 10.1097/MCG.0000000000002164.

9. Lee TY, Hsieh TC, Sung HC, Chen WL. Internet-Delivered Cognitive Behavior Therapy for Young Taiwanese Female Nursing Students with Irritable Bowel Syndrome-A Cluster Randomized Controlled Trial. Int J Environ Res Public Health. 2019 Feb 27;16(5). PMID: 30818837. doi: 10.3390/ijerph16050708.

10. Lindfors P, Axelsson E, Engstrand K, Störsrud S, Jerlstad P, Törnblom H, et al. Online Education Is Non-Inferior to Group Education for Irritable Bowel Syndrome: a Randomized Trial and Patient Preference Trial. Clin Gastroenterol Hepatol. 2020;19(4):743‐51.e1. PMID: CN-02099449. doi: 10.1016/j.cgh.2020.04.005.

11. Ljótsson B, Falk L, Vesterlund AW, Hedman E, Lindfors P, Rück C, et al. Internet-delivered exposure and mindfulness based therapy for irritable bowel syndrome - A randomized controlled trial. Behav Res Ther. 2010;48(6):531-9. PMID: 20362976. doi: 10.1016/j.brat.2010.03.003.

12. Ljotsson B, Andersson G, Andersson E, Hedman E, Lindfors P, Andreewitch S, et al. Acceptability, effectiveness, and cost-effectiveness of internet-based exposure treatment for irritable bowel syndrome in a clinical sample: a randomized controlled trial. BMC Gastroenterol. 2011 Oct 12;11. PMID: WOS:000296617700001. doi: 10.1186/1471-230x-11-110.

13. Ljótsson B, Hedman E, Andersson E, Hesser H, Lindfors P, Hursti T, et al. Internet-delivered exposure-based treatment vs. stress management for irritable bowel syndrome: a randomized trial. The American Journal of Gastroenterology. 2011;106(8):1481-91. PMID: 21537360. doi: 10.1038/ajg.2011.139.

14. Ljótsson Bn, Hesser H, Andersson E, Lackner JM, El Alaoui S, Falk L, et al. Provoking symptoms to relieve symptoms: A randomized controlled dismantling study of exposure therapy in irritable bowel syndrome. BEHAVIOUR RESEARCH AND THERAPY. 2014. PMID: 24584055. doi: 10.1016/j.brat.2014.01.007.

15. Owusu JT, Sibelli A, Moss-Morris R, van Tilburg MAL, Levy RL, Oser M. A pilot feasibility study of an unguided, internet-delivered cognitive behavioral therapy program for irritable bowel syndrome. Neurogastroenterol Motil. 2021 Nov;33(11):e14108. PMID: 33745228. doi: 10.1111/nmo.14108.

16. Tayama J, Hamaguchi T, Koizumi K, Yamamura R, Okubo R, Kawahara J-i, et al. Efficacy of an eHealth self-management program in reducing irritable bowel syndrome symptom severity: a randomized controlled trial. Scientific Reports. 2024 Jan 3;14(1). PMID: WOS:001145989400090. doi: 10.1038/s41598-023-50293-z.

17. Zargar F, Fahim A, Nikgoftar N, Tarrahi MJ. Comparing the effect of internet-delivered short-term progressive muscle relaxation and psychoeducation on mindful ability, visceral hypersensitivity and symptoms of patients with irritable bowel syndrome. J Educ Health Promot. 2023;12:259. PMID: 37727438. doi: 10.4103/jehp.jehp_1734_22.
